# Dietary Choline and Betaine Intake and 2-year Changes in Cognitive Function in Older Adults With Overweight or Obesity and Metabolic Syndrome: A Prospective Cohort Analysis

**DOI:** 10.1016/j.ajcnut.2026.101265

**Published:** 2026-03-11

**Authors:** Héctor Vázquez-Lorente, José María Manzanares-Errazu, Nancy Babio, Miguel Ruiz-Canela, Dolores Corella, Javi Hernando, José Alfredo Martínez, Ángel M Alonso-Gómez, Julia Wärnberg, Jesús Vioque, Dora Romaguera, José López-Miranda, Ramon Estruch, Francisco J Tinahones, Víctor Urbano-Fernández, Lluís Serra-Majem, Naomi Cano-Ibáñez, Josep A Tur, Vicente Martín Sánchez, Xavier Pintó, Miguel Delgado-Rodríguez, Pilar Matía-Martín, Josep Vidal, Clotilde Vázquez, Emili Ros, Fernando Fernández-Aranda, Estefanía Toledo, Liliana Gutiérrez-Carrasquilla, José V Sorlí, María Dolores Zomeño, Antonio Garcia-Rios, Alejandro Oncina-Canovas, Raquel Cueto-Galán, María Angeles Zulet, Lara Prohens, Rosa Casas, María Durán-Luque, Lucas Tojal-Sierra, Víctor Simón-Frapolli, Zenaida Vázquez-Ruiz, Rebeca Fernández-Carrión, Olga Castañer, Aquiles Lozano Rodriguez-Mancheño, Alberto Asencio, Ana García-Arellano, Montse Fitó, Lidia Daimiel, Jordi Salas-Salvadó

**Affiliations:** 1 Departament de Bioquímica i Biotecnologia, Alimentació, Nutrició, Desenvolupament i Salut Mental ANUT-DSM, Universitat Rovira i Virgili, Reus, Spain; 2 Institut d'Investigació Sanitària Pere Virgili (IISPV), Reus, Spain; 3 Centro de Investigación Biomédica en Red Fisiopatología de la Obesidad y la Nutrición (CIBEROBN), Institute of Health Carlos III, Madrid, Spain; 4 Hospital Universitari Sant Joan de Reus, Universitat Rovira i Virgili, IISPV, Alimentació, Nutrició, Desenvolupament i Salut Mental ANUT-DSM, Reus, Spain; 5 Department of Preventive Medicine and Public Health, Instituto de Investigación Sanitaria de Navarra (IdiSNA), University of Navarra, Pamplona, Spain; 6 Department of Preventive Medicine, University of Valencia, Valencia, Spain; 7 Unit of Cardiovascular Risk and Nutrition, Hospital del Mar Research Institute (IMIM), Barcelona, Spain; 8 Department of Nutrition, Food Sciences, and Physiology, Center for Nutrition Research, University of Navarra, Pamplona, Spain; 9 Precision Nutrition and Cardiometabolic Health Program, IEA Food, CEI UAM + CSIC, Madrid, Spain; 10 Departamento de Medicina y Endocrinología, Universidad de Valladolid, Valladolid, Spain; 11 Bioaraba Health Research Institute, Cardiovascular, Respiratory and Metabolic Area, Osakidetza Basque Health Service, Araba University Hospital, University of the Basque Country UPV/EHU, Vitoria-Gasteiz, Spain; 12 EpiPHAAN Research Group, School of Health Sciences, University of Málaga – Instituto de Investigación Biomédica en Málaga (IBIMA), Málaga, Spain; 13 CIBER de Epidemiología y Salud Pública (CIBERESP), Instituto de Salud Carlos III (ISCIII), Madrid, Spain; 14 Instituto Instituto de Investigación Sanitaria y Biomédica de Alicante, ISABIAL, Alicante, Spain; 15 Unidad de Epidemiología de la Nutrición, Universidad Miguel Hernández (UMH), Alicante, Spain; 16 Health Research Institute of the Balearic Islands (IdISBa), Palma de Mallorca, Spain; 17 Department of Internal Medicine, Maimonides Biomedical Research Institute of Cordoba (IMIBIC), Reina Sofia University Hospital, University of Cordoba, Cordoba, Spain; 18 Department of Internal Medicine, Institut d'Investigacions Biomèdiques August Pi Sunyer (IDIBAPS), Hospital Clinic, University of Barcelona, Barcelona, Spain; 19 Institut de Recerca en Nutrició i Seguretat Alimentaria (INSA-UB), University of Barcelona, Barcelona, Spain; 20 Department of Endocrinology, Virgen de la Victoria Hospital, Instituto de Investigación Biomédica de Málaga (IBIMA), University of Málaga, Málaga, Spain; 21 Department of Family Medicine, Research Unit, Distrito Sanitario Atención Primaria Sevilla, Sevilla, Spain; 22 Research Institute of Biomedical and Health Sciences (IUIBS), University of Las Palmas de Gran Canaria & Centro Hospitalario Universitario Insular Materno Infantil (CHUIMI), Canarian Health Service, Las Palmas de Gran Canaria, Spain; 23 Department of Preventive Medicine and Public Health, University of Granada, Granada, Spain; 24 Institute for Biosanitary Research ibs.GRANADA, Granada, Spain; 25 Research Group on Community Nutrition & Oxidative Stress, University of Balearic Islands, Palma de Mallorca, Spain; 26 Institute of Biomedicine (IBIOMED), University of León, León, Spain; 27 Lipids and Vascular Risk Unit, Internal Medicine, Hospital Universitario de Bellvitge-IDIBELL, Hospitalet de Llobregat – Barcelona, Barcelona, Spain; 28 Division of Preventive Medicine, Faculty of Medicine, University of Jaén, Jaén, Spain; 29 Department of Endocrinology and Nutrition, Instituto de Investigación Sanitaria Hospital Clínico San Carlos (IdISSC), Madrid, Spain; 30 CIBER Diabetes y Enfermedades Metabólicas (CIBERDEM), Instituto de Salud Carlos III (ISCIII), Madrid, Spain; 31 Department of Endocrinology, Institut d`Investigacions Biomèdiques August Pi Sunyer (IDIBAPS), Hospital Clinic, University of Barcelona, Barcelona, Spain; 32 Department of Endocrinology and Nutrition, Hospital Fundación Jimenez Díaz, Instituto de Investigaciones Biomédicas IISFJD, University Autonoma, Madrid, Spain; 33 Lipid Clinic, Department of Endocrinology and Nutrition, Institut d'Investigacions Biomèdiques August Pi Sunyer (IDIBAPS), Hospital Clínic, Barcelona, Spain; 34 Psychoneurobiology of Eating and Addictive Behaviors Group, Institut d'Investigació Biomédica de Bellvitge (IDIBELL), Barcelona, Spain; 35 Department of Clinical Psychology, University Hospital of Bellvitge and University of Barcelona, Barcelona, Spain; 36 Faculty of Health Science, Universidad Internacional de La Rioja (UNIR), Logroño, La Rioja, Spain; 37 Centro Atención Primaria de Mutxamel, Consellería de Sanitat, Generalitat Valenciana, Alicante, Spain; 38 Hospital Universitario de Navarra, Servicio de Urgencias, Pamplona, Spain; 39 Nutritional Control of the Epigenome Group, Precision Nutrition and Obesity Program, IMDEA Food, CEI UAM + CSIC, Madrid, Spain; 40 Departamento de Ciencias Farmacéuticas y de la Salud, Faculty de Farmacia, Universidad San Pablo-CEU, CEU Universities, Boadilla del Monte, Spain

**Keywords:** choline, betaine, cognition, cognitive decline, cognitive function, ageing, older adults

## Abstract

**Background:**

Dietary choline and betaine may protect against cognitive decline.

**Objectives:**

To examine longitudinal associations between dietary choline and betaine intake and 2-y cognitive changes in older adults.

**Methods:**

This prospective cohort study is a secondary analysis nested within the PREDIMED-Plus trial. Participants included 6610 older adults aged 55–75 y with metabolic syndrome. Dietary choline and betaine intake were estimated at baseline, 1, and 2 y using a validated 143-item food frequency questionnaire. Cumulative averages were calculated. Cognitive function was assessed at baseline and 2 y using 5 composite scores based on 8 neuropsychological tests covering global cognition, general cognition, attention, executive function, and language. Multivariable-adjusted linear regression models evaluated associations between energy-adjusted cumulative average intakes and 2-y cognitive changes.

**Results:**

Over a median follow-up of 2 y (interquartile range: 1.95–2.05), each 1 mg/d higher energy-adjusted cumulative average dietary choline intake was associated with slower decline in attention [*β* = 5.20 × 10^−4^; 95% confidence interval (CI): 1.61 × 10^−4^, 8.79 × 10^−4^; *P* = 0.005] and beneficial changes in language (*β* = 3.79 × 10^−4^; 95% CI: 0.62 × 10^−4^, 6.95 × 10^−4^; *P* = 0.019). Participants in the highest choline tertile showed greater 2-y improvements in attention (*β* = 7.50 × 10^−2^; 95% CI: 2.12 × 10^−2^, 12.88 × 10^−2^; *P*-trend = 0.006) and language (*β* = 5.82 × 10^−2^; 95% CI: 1.04 × 10^−2^, 10.59 × 10^−2^; *P*-trend = 0.016) compared with the lowest tertile. Similarly, each 1 mg/d higher betaine intake was associated with more favorable changes in executive function (*β* = 7.48 × 10^−4^; 95% CI: 1.71 × 10^−4^, 13.20 × 10^−4^; *P* = 0.011) and language (*β* = 9.13 × 10^−4^; 95% CI: 2.96 × 10^−4^, 15.31 × 10^−4^; *P* = 0.004). Participants in the highest betaine tertile also exhibited greater 2-y improvements in language (*β* = 4.71 × 10^−2^; 95% CI: 0.25 × 10^−2^, 9.17 × 10^−2^; *P*-trend = 0.036) compared with the lowest tertile.

**Conclusions:**

Higher dietary choline and betaine intake were associated with modest short-term improvements in cognitive performance over 2 y in older adults. Longer-term studies are warranted.

This trial was registered at www.isrctn.com as ISRCTN89898870.

## Introduction

Cognitive impairment, Alzheimer’s disease, and other dementias are major age-related public health priorities [1] because of their high prevalence, lack of curative treatments, and projected substantial increase in developed countries by 2050 [2]. Older adults are vulnerable to cognitive decline, a risk further heightened by overweight or obesity and metabolic syndrome [3], whose associated metabolic and vascular alterations may accelerate neurodegeneration [4].

Lifestyle factors, particularly diet, play a pivotal role in determining cognitive health in older adults [5]. Primarily derived from animal-based foods (e.g., eggs, chicken, fish, beef, and dairy products) [6], choline has emerged as a nutrient of interest because of its role in neurobiological mechanisms, including phosphatidylcholine metabolism linked to cognitive decline [7,8]. However, evidence on whether dietary choline intake affects cognitive performance in adults remains limited and inconsistent. Few community-based cross-sectional [[9], [10], [11], [12], [13]] and longitudinal studies have examined this association, results being mixed and based in individual cognitive performance tests [[14], [15], [16]]. These inconsistencies may stem from differences in study populations, dietary and cognitive performance assessment methodologies, and variability in dietary patterns across cohorts [17].

Betaine, a metabolite derived from choline metabolism, is a nutrient found in various foods such as whole grains, spinach, beets, and shellfish. It can be obtained through the diet or produced endogenously from choline [18]. Inadequate dietary intake of betaine has been implicated in cognitive health disturbances [19]. Although animal studies have demonstrated promising beneficial effects of betaine on cognitive health [[20], [21], [22]], it remains unclear to date if the neuroprotective effects of choline observed in animal studies translate to humans. In this line, observational human studies investigating this association reported inconclusive findings for individual tests encompassing cognitive performance in a young cohort of premenopausal females [8]. Moreover, maternal dietary betaine intake showed no relationship with improved cognitive performance domains in their children [23], highlighting the need for further research in older populations where cognitive decline is more pronounced.

Prior studies have mainly focused on individual cognitive tests, despite evidence that composite cognitive scores provide greater reliability and sensitivity for detecting subtle cognitive changes in older adults, particularly in populations at high risk of cognitive decline [24]. The PREDIMED-Plus study provides an opportunity to address this gap by examining a large, well-characterized cohort of older adults who are cognitively unimpaired at baseline but present overweight or obesity and metabolic syndrome, well-established modifiable risk factors for cognitive decline and dementia [25]. This setting enables the assessment of potentially protective dietary factors during a critical window before clinically overt cognitive impairment, when cognitive trajectories may still be modifiable [26]. Accordingly, this study aimed to evaluate the longitudinal associations between dietary choline and betaine intake and 2-y changes in cognitive performance, hypothesizing that higher intakes may be associated with more favorable cognitive changes over time.

## Methods

### Study design

This is an observational prospective cohort study nested under the framework of the PREDIMED-Plus trial [27]. Briefly, PREDIMED-Plus is a multicenter, parallel-group, randomized, single-blind clinical trial evaluating the long-term effects of a lifestyle intervention including an energy-reduced Mediterranean diet (erMedDiet), physical activity promotion, and behavioral support for weight loss (intervention group) compared with general ad libitum Mediterranean diet recommendations (control group) on primary cardiovascular disease prevention and total body weight loss. Neither study arm included specific guidance regarding choline- or betaine-rich foods. Further details regarding the trial protocol can be accessed at https://www.predimedplus.com/, and in previously published sources [28,29]. Ethical approval was obtained from all participating centers, and written informed consent was obtained from all participants. The trial was registered in 2014 at www.isrctn.com/ISRCTN89898870.

### Participants

**FIGURE 1:**
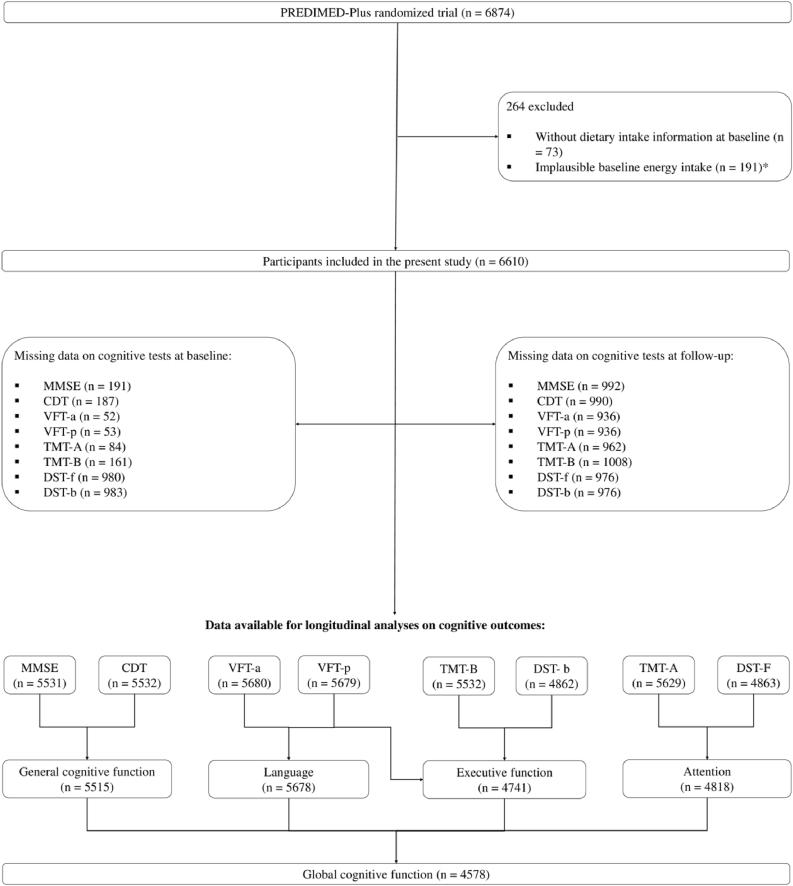
Flowchart of the study population. CDT, Clock Drawing Test; DST-b, Digit Span test backward; DST-f, Digit Span test forward; MMSE, Mini-Mental State Examination; TMT-A, Trail Making Test Part A; TMT-B, Trail Making Test Part B; VFT-a, Verbal Fluency tasks semantical; VFT-p, Verbal Fluency tasks phonological. ∗Daily energy intakes for males <800 kcal or >4000 kcal and females <500 kcal or >3500 kcal.

The study enrolled community-dwelling adults aged 55–75 y with overweight or obesity (BMI ranging from 27 to 40 kg/m^2^) who met ≥3 criteria for metabolic syndrome [30]. Exclusion criteria were based on *1*) unwillingness to give written informed consent, *2*) institutionalization, *3*) pre-existing cardiovascular diseases, psychiatric disorders, bowel diseases, or clinical diagnosis of mental or neurological disease, including dementia or other relevant brain disorders, *4*) weight loss medication use, and *5*) inability to follow the intervention based on religious purposes, food allergies, or intolerances. From October 2013 to December 2016, a total of 6874 eligible participants were randomly assigned in a 1:1 ratio to either the intervention group or the control group. The randomization procedure was blinded to all staff members and principal investigators. For participant couples sharing the same household, randomization was done by cluster, with the couple as the unit of randomization. For the purposes of this study, participants not completing dietary questionnaires, and reporting energy intakes outside predefined limits (<800 to ≥4000 kcal/d for males, <500 to ≥3500 kcal/d for females) at baseline were excluded [31]. A total of 6610 participants were finally included in the study (Figure 1).

### Exposure: dietary choline and betaine intake

Trained dietitians conducted face-to-face interviews with participants to assess their dietary habits using a validated 143-item food frequency questionnaire (FFQ) [32]. Participants were asked to define the frequency of consumption of each food item within a usual year. Nine options for frequency of consumption were given, ranging from “never or hardly ever” to “more than six times a day.” Food intake (g/d) was then estimated by multiplying the portion size by the frequency of consumption and dividing by 7 or by 30 for weekly or monthly consumed food items, respectively. Nutrient estimates were calculated from all items in the FFQ responses that contributed to nutrient intake, by using Spanish food composition tables [33]. To estimate dietary choline and betaine intake, we used USDA databases from 2015 to 2018 and the website https://nutritiondata.self.com/ and multiplied gp/d of each food item by the amount of choline or betaine per gram of food. Assessment of choline intake through an FFQ has been previously validated in the Framingham Offspring Study [34].

Dietary choline and betaine intake was assessed at baseline, 1 y, and 2 y of follow-up. The energy-adjusted cumulative average of dietary choline and betaine intake over these time points was subsequently calculated by summing the reported dietary choline and betaine intake from each time point and dividing by the number of assessments available up to that moment, and using the residual method [35] to better represent long-term intake, reduce within-person variation and measurement errors. Participants were additionally categorized into tertiles of energy-adjusted cumulative average dietary choline and betaine intake, respectively, over time.

### Outcome: changes in cognitive function

Cognitive function evaluation was conducted by trained personnel both at baseline and at 2-y follow-up assessment following standardized protocols and were not blinded to participants’ intervention group assignment. A battery of 8 neuropsychological tests, validated specifically for the Spanish population, was administered via individual interviews. These tests comprised the Mini-Mental State Examination (MMSE), the Clock Drawing Test, the Verbal Fluency Tests, the forward and backward versions of the Digit Span Test from the Wechsler Adult Intelligence Scale-III, and the Trail Making Test parts A. Detailed descriptions of these neuropsychological assessments can be found elsewhere [36] and in Supplemental Methods. Each cognitive test was administered at baseline and at the 2-y follow-up period and transformed to a baseline-referenced z-score using the baseline mean and SD. This approach anchors follow-up performance to the baseline distribution and facilitates comparability across tests with different score ranges; however, it does not completely eliminate potential retesting-related gains. Moreover, all participants followed identical testing schedules, any residual practice/learning effects should be broadly similar across exposure tertiles and are unlikely to account for the observed between-tertile differences in change. Therefore, the final interpretation of 2-y change considers 3 related concepts: *1*) practice/learning effects (systematic gains because of familiarity with test procedures), *2*) test–retest variability (random within-person fluctuation and measurement noise), and *3*) test–retest reliability (stability of scores across administrations). Change scores were computed as the difference between follow-up and baseline z-scores [[37], [38], [39]].

Composite measures for 4 cognitive composite scores (general cognitive function, executive function, attention, and language), as well as a global assessment of cognitive function, were computed for each participant. The composite cognitive assessments were determined by aggregating or deducting individual test z-scores based on whether a higher score indicates superior or inferior cognitive function, respectively, as detailed in Supplemental Table 1 and Supplemental Methods. Following this procedure, the resulting 5 composite scores were further standardized to z-scores using the mean and SD values from baseline composite score data. Composite scores generally increase measurement stability relative to single-test outcomes, improving sensitivity to modest longitudinal changes. The changes observed in these 5 composite scores over the 2-y period constituted the primary outcome of interest in this study.

### Covariate assessments

Sociodemographic and lifestyle information encompassing age, sex, education level, marital status, and smoking habits were gathered at baseline by administered questionnaires. Baseline physical activity was estimated utilizing a validated Spanish short version of the Minnesota Leisure Time Physical Activity Questionnaire (the REGICOR questionnaire) [40]. Personal medical history, encompassing conditions such as baseline type 2 diabetes, hypertension, and hypercholesterolemia, as well as medication usage, were either self-reported or extracted from medical records. Depressive symptomatology was evaluated at baseline using the Beck Depression Inventory-II [41,42]. Anthropometric variables, such as weight and height, were assessed at baseline using calibrated scales and wall-mounted stadiometers, respectively. BMI was calculated as weight (kg)/height (m^2^). Alcohol consumption was collected at baseline using the same FFQ used to assess dietary choline and betaine intake. Energy-adjusted alcohol consumption was obtained using the residual method [35]. Adherence to the erMedDiet was assessed at baseline, 1, and 2 y of follow-up via a validated 17-point scale questionnaire [17-item energy-restricted Mediterranean Adherence Screener (erMEDAS)], where compliance with each of the 17 items was scored with 1 point; therefore, the total erMEDAS score was ranged between 0 and 17, with 0 meaning null adherence and 17 meaning maximum adherence [43]. Cumulative average adherence to the erMedDiet was subsequently obtained.

### Statistical analyses

As this study is a secondary and prospective cohort analysis nested within the PREDIMED-Plus randomized controlled trial, the sample size was defined by the number of participants with available dietary and cognitive data at baseline and at the 2-y follow-up, rather than by an a priori power calculation specific to dietary choline or betaine intake. The large size of the PREDIMED-Plus cohort ensures sufficient precision to evaluate modest longitudinal associations. Analyses included all participants with available dietary exposure and cognitive outcome data at baseline. For longitudinal analyses, participants were included if data for the specific cognitive outcome of interest were available at the 2-y follow-up assessment. Missing follow-up data were retained as missing and were not imputed or excluded, as illustrated in the study flowchart (Figure 1).

Baseline characteristics of the study cohort were presented both in overall and categorized by tertiles of energy-adjusted cumulative average dietary choline and betaine intake over time as means ± SDs for continuous variables, and numbers (percentages) for categorical variables. One-way analysis of variance was employed for continuous variables, whereas chi-square tests were utilized for categorical variables. For variables with statistically significant overall differences across tertiles, post hoc pairwise comparisons between tertiles were conducted.

The longitudinal associations between energy-adjusted cumulative average dietary choline and betaine intake (exposures) and changes observed in cognitive function measurements over a 2-y period (outcome) were investigated through multivariable linear regression models. These models were adjusted for potential confounders pertinent to cognitive function. A basic model was adjusted for the respective baseline cognitive function score, age (y), and sex (male or female). The intervention group (control or intervention), recruiting center size (<200, 200–300, 300–450 or >450), education level (primary or less, secondary, or college), marital status (single, divorced, or separated, married, or widower), BMI, physical activity (metabolic equivalents in min/d), smoking status (never, former, or current), baseline alcohol consumption in g/d (and adding the quadratic term), baseline presence of depressive symptomatology (yes or no), type 2 diabetes (yes or no), hypertension (yes or no), hypercholesterolemia (yes or no), and cumulative average erMEDAS (low, median, or high) were additionally included as covariates in a multivariable fully adjusted model. Robust variance estimators were utilized in all models to accommodate intracluster correlations, as couples from the same household were randomized together. Results were presented as *β*-coefficients along with their corresponding 95% confidence intervals (CIs). Restricted cubic splines were checked to assess potential nonlinear associations using cumulative average energy-adjusted dietary choline and betaine intakes as continuous variables. We used 3 knots (10th, 50th, and 90th percentiles) and evaluated nonlinearity with a Wald test of the spline’s nonlinear terms [44].

A priori interaction analyses were conducted for cognitive performance composite scores by baseline categories of age (<65 or ≥65 y), sex (male or female), intervention group (control or intervention), educational level (low or high), and type 2 diabetes prevalence (yes or no), using the likelihood ratio test by comparing models with and without the multiplicative interaction term between these factors and energy-adjusted cumulative average dietary choline and betaine intake within fully adjusted models. Results were further adjusted for multiple comparisons using the Benjamini–Hochberg (BH) method [45].

All statistical analyses were conducted with Stata/SE version 14.2 (StataCorp LLC) using the PREDIMED-Plus study dataset updated to 19 December 2023. All graphs were plotted using GraphPad Prism software v.9.0 (GraphPad Software). Statistical significance was defined as a 2-tailed *P* value <0.05.

## Results

TABLE 1, TABLE 2 show the baseline characteristics of the overall study population and by tertiles of energy-adjusted cumulative average dietary choline and betaine intake over time, respectively. Energy-adjusted cumulative average daily dietary choline intake ranged from 351.8 ± 33.0 mg/d in the lowest tertile to 493.3 ± 42.6 mg/d in the highest tertile (overall mean: 421.8 ± 66.2 mg/d), whereas energy-adjusted cumulative average daily dietary betaine intake ranged from 82.0 ± 13.9 mg/d in the lowest tertile to 148.2 ± 22.7 mg/d in the highest tertile (overall mean: 114.1 ± 31.4 mg/d). Additional information regarding all baseline variables of the study is shown in TABLE 1, TABLE 2, respectively.

**TABLE 1:** Baseline characteristics of the PREDIMED-Plus participants both in overall and by tertiles of energy-adjusted cumulative average dietary choline intake.

Characteristic	Total population	Categories of dietary choline intake			*P* value^1^
		1st tertile	2nd tertile	3rd tertile	
Choline, mean (SD) (mg/d)	421.8 ± 66.2	351.8 ± 33.0^a^	420.1 ± 15.5^b^	493.3 ± 42.6^c^	0.001^2^
Sociodemographic variables					
Age, mean (SD) (y)	65.0 ± 4.9	64.7 ± 5.1^a^	65.1 ± 4.9^b^	65.2 ± 4.8^c^	0.001^2^
Female, *N* (%)	3201 (48.4)	786 (35.7)^a^	1111 (50.4)^b^	1304 (59.2)^c^	0.001^2^
Education level, *N* (%)					
Primary or less	3250 (49.2)	1100 (49.9)	1088 (49.4)	1062 (48.2)	
Secondary	1907 (28.9)	618 (28.0)	647 (29.4)	642 (29.1)	0.653
College	1453 (22.0)	486 (22.1)	468 (21.2)	499 (22.7)	
Marital status, *N* (%)					
Single, divorced, or separated	851 (12.9)	307 (13.9)^a^	270 (12.3)^b^	274 (12.4)^b^	
Married	5072 (76.7)	1687 (76.5)^a^	1716 (77.9)^b^	1669 (75.8)^c^	0.041^2^
Widower	687 (10.4)	210 (9.5)^a^	217 (9.9)^a^	260 (11.8)^b^	
Disease presence or medication usage at recruitment					
Type 2 diabetes, *N* (%)	2070 (31.3)	623 (28.3)^a^	719 (32.6)^b^	728 (33.1)^b^	0.001^2^
Hypertension, *N* (%)	5511 (83.4)	1842 (83.6)	1823 (82.8)	1846 (83.8)	0.668
Hypercholesterolemia, *N* (%)	4572 (69.2)	1525 (69.2)	1510 (68.5)	1537 (69.8)	0.902
Depressive symptomatology, *N* (%)	1359 (20.6)	434 (19.7)	451 (20.5)	474 (21.5)	0.323
Anthropometric variables					
BMI, mean (SD) (kg/m^2^)	32.5 ± 3.4	32.3 ± 3.4^a^	32.5 ± 3.4^b^	32.8 ± 3.4^c^	0.001^2^
Lifestyle variables					
Physical activity, mean (SD) (METs/min/d)	351.8 ± 329.1	350.9 ± 343.5	350.5 ± 323.2	353.8 ± 320.4	0.937
Smoking status, *N* (%)					
Current smoker	819 (12.4)	349 (15.8)^a^	265 (12.0)^b^	205 (9.3)^c^	0.001^2^
Former smoker	2857 (43.2)	1028 (46.6)^a^	943 (42.8)^b^	886 (40.2)^c^	
Never smoker	2934 (44.4)	827 (37.6)^a^	995 (45.2)^b^	1112 (50.5)^c^	
Alcohol intake, mean (SD) (g/d)	11.0 (15.0)	15.2 (18.1)^a^	9.9 (13.6)^b^	7.9 (11.6)^c^	0.001^2^
erMEDAS, *N* (%)					
Low	1576 (23.8)	687 (31.2)^a^	538 (24.4)^b^	351 (15.9)^c^	0.001^2^
Medium	2647 (40.1)	917 (41.6)^a^	875 (39.7)^b^	855 (38.8)^c^	
High	2387 (36.1)	600 (27.2)^a^	790 (35.9)^b^	997 (45.3)^c^	
Cognitive function assessment					
Global Cognitive Function^3^ (*n* = 4557)	0.2 ± 1.0	0.3 ± 1.0	−0.4 ± 1.0	−0.1 ± 1.0	0.979
General Cognitive Function^3^ (*n* = 5488)	0.1 ± 1.0	−1.4 ± 1.0	3.5 ± 1.0	−2.3 ± 1.0	0.169
Executive Function^3^ (*n* = 4720)	0.2 ± 1.0	−0.1 ± 1.0	−1.6 ± 1.0	1.9 ± 1.0	0.616
Attention^3^ (*n* = 4797)	0.1 ± 1.0	2.1 ± 1.0	−1.9 ± 1.0	−0.3 ± 1.0	0.513
Language^3^ (*n* = 5651)	0.1 ± 1.0	0.3 ± 1.0	−1.9 ± 1.0	1.7 ± 1.0	0.549

Data are presented as *n* (%) or mean ± SD for categorical and continuous variables, respectively.

Abbreviations: CI, confidence interval; erMEDAS, energy-restricted Mediterranean Adherence Screener; MET, metabolic equivalent.

1 *P* value for differences between categories of energy-adjusted cumulative average dietary choline intake was calculated by Pearson’s Chi-square test or one-way analysis of variance, as appropriate. Superscript letters (a, b, c) indicate results of post hoc pairwise comparisons with Bonferroni adjustment between tertiles. Within each row, tertiles sharing the same letter are not significantly different, whereas tertiles with different letters are significantly different.

2 Indicates significant values (*P* < 0.05).

3 Mean values are presented as multiples of 10^−2^ (×10^−2^).

**TABLE 2:** Baseline characteristics of the PREDIMED-Plus participants both in overall and by tertiles of energy-adjusted cumulative average dietary betaine intake

Characteristic	Total population	Categories of dietary betaine intake			*P* value^1^
		1st tertile	2nd tertile	3rd tertile	
Betaine, mean (SD) (mg/d)	114.1 ± 31.4	82.0 ± 13.9^a^	112.0 ± 7.2^b^	148.2 ± 22.7^c^	0.001^2^
Sociodemographic variables					
Age, mean (SD) (y)	65.0 ± 4.9	64.8 ± 4.9^a^	64.9 ± 4.9^b^	65.2 ± 4.9^c^	0.019^2^
Female, *N* (%)	3201 (48.4)	936 (42.5)^a^	1111 (50.4)^b^	1154 (52.4)^c^	0.001^2^
Education level, *N* (%)					
Primary or less	3250 (49.2)	1001 (45.4)^a^	1103 (50.1)^b^	1146 (52.0)^c^	
Secondary	1907 (28.9)	659 (29.9)^a^	635 (28.8)^b^	613 (27.8)^c^	0.001^2^
College	1453 (22.0)	544 (24.7)^a^	465 (21.1)^b^	444 (20.2)^c^	
Marital status, *N* (%)					
Single, divorced, or separated	851 (12.9)	302 (13.7)^a^	295 (13.4)^a^	254 (11.5)^b^	
Married	5072 (76.7)	1700 (77.1)^a^	1667 (75.7)^b^	1705 (77.4)^a^	0.040^2^
Widower	687 (10.4)	202 (9.2)^a^	241 (10.9)^b^	244 (11.1)^b^	
Disease presence or medication usage at recruitment					
Type 2 diabetes, *N* (%)	2070 (31.3)	609 (27.6)^a^	703 (31.9)^b^	758 (34.4)^c^	0.001^2^
Hypertension, *N* (%)	5511 (83.4)	1798 (81.6)^a^	1836 (83.3)^b^	1877 (85.2)^c^	0.001^2^
Hypercholesterolemia, *N* (%)	4572 (69.2)	1472 (66.8)^a^	1533 (69.6)^b^	1567 (71.1)^c^	0.008^2^
Depressive symptomatology, *N* (%)	1359 (20.6)	439 (19.9)^a^	418 (19.0)^b^	502 (22.8)^c^	0.005^2^
Anthropometric variables					
BMI, mean (SD) (kg/m^2^)	32.5 ± 3.4	32.3 ± 3.4^a^	32.5 ± 3.4^b^	32.8 ± 3.5^c^	0.001^2^
Lifestyle variables					
Physical activity, mean (SD) (METs/min/d)	351.8 ± 329.1	373.1 ± 336.6^a^	347.1 ± 331.8^b^	335.2 ± 317.7^b^	0.001^2^
Smoking status, *N* (%)					
Current smoker	819 (12.4)	310 (14.0)^a^	265 (12.0)^b^	244 (11.1)^c^	0.017^2^
Former smoker	2857 (43.2)	976 (44.3)^a^	931 (42.3)^b^	950 (43.1)^c^	
Never smoker	2934 (44.4)	918 (41.7)^a^	1007 (45.7)^b^	1009 (45.8)^c^	
Alcohol intake, mean (SD) (g/d)	11.0 (15.0)	14.9 (17.7)^a^	9.6 (13.2)^b^	8.6 (12.8)^b^	0.001^2^
erMEDAS, *N* (%)					
Low	1576 (23.8)	507 (23.0)^a^	514 (23.3)^b^	555 (25.2)^c^	0.002^2^
Medium	2647 (40.1)	837 (38.0)^a^	893 (40.5)^b^	917 (41.6)^c^	
High	2387 (36.1)	860 (39.0)^a^	796 (36.1)^b^	731 (33.2)^c^	
Cognitive function assessment					
Global Cognitive Function^3^ (*n* = 4557)	0.2 ± 1.0	7.6 ± 1.0^a^	−3.5 ± 1.0^b^	−5.1 ± 1.0^b^	0.001^2^
General Cognitive Function^3^ (*n* = 5488)	0.1 ± 1.0	9.0 ± 1.0^a^	−3.7 ± 1.0^b^	−6.0 ± 1.0^b^	0.001^2^
Executive Function^3^ (*n* = 4720)	0.2 ± 1.0	6.8 ± 1.0^a^	−3.2 ± 1.0^b^	−4.3 ± 1.0^b^	0.002^2^
Attention^3^ (*n* = 4797)	0.1 ± 1.0	6.1 ± 1.0^a^	−2.2 ± 1.0^b^	−4.6 ± 1.0^b^	0.005^2^
Language^3^ (*n* = 5651)	0.1 ± 1.0	6.4 ± 1.0^a^	−1.3 ± 1.0^b^	−5.5 ± 1.0^b^	0.001^2^

Data are presented as *n* (%) or mean ± SD for categorical and continuous variables, respectively.

Abbreviations: CI, confidence interval; erMEDAS, energy-restricted Mediterranean Adherence Screener; MET, metabolic equivalent.

1 *P* value for differences between categories of energy-adjusted cumulative average dietary betaine intake was calculated by Pearson’s Chi-square test or one-way analysis of variance, as appropriate. Superscript letters (a, b, c) indicate results of post hoc pairwise comparisons with Bonferroni adjustment between tertiles. Within each row, tertiles sharing the same letter are not significantly different, whereas tertiles with different letters are significantly different.

2 Indicates significant values (*P* < 0.05).

3 Mean values are presented as multiples of 10^−2^ (×10^−2^).

Over a median follow-up of 2 y (IQR: 1.95–2.05), improvements in global and general cognitive function, executive function and language, and a decline in attention composite scores were noted overall, and across tertiles of energy-adjusted cumulative average dietary choline intake (Supplemental Table 2) and energy-adjusted cumulative average dietary betaine intake (Supplemental Table 3). Additional information on changes in the battery of 8 individual cognitive performance tests is presented in Supplemental Tables 4 and 5 for dietary choline and betaine, respectively.

TABLE 3, TABLE 4 display the longitudinal associations (*β* coefficients and 95% CI) between energy-adjusted cumulative average dietary choline and betaine intake and changes in cognitive function over a median follow-up of 2 y (IQR: 1.95–2.05) period. Results from multivariable-adjusted models show significant positive associations between energy-adjusted cumulative average dietary choline and betaine intake and 2-y beneficial changes in cognitive function. Restricted cubic spline analyses (3 knots) showed no evidence of departure from linearity (*P*-nonlinearity >0.05 for all associations); therefore, results are presented using linear terms. In particular, each 1 mg/d higher energy-adjusted cumulative average dietary choline intake was associated with slower decline in attention (*β* = 5.20 × 10^−4^; 95% CI: 1.61 × 10^−4^, 8.79 × 10^−4^; *P* = 0.005) and beneficial changes in language (*β* = 3.79 × 10^−4^; 95% CI: 0.62 × 10^−4^, 6.95 × 10^−4^; *P* = 0.019). Participants in the highest choline tertile showed greater 2-y improvements in attention (*β* = 7.50 × 10^−2^; 95% CI: 2.12 × 10^−2^, 12.88 × 10^−2^; *P*-trend = 0.006) and language (*β* = 5.82 × 10^−2^; 95% CI: 1.04 × 10^−2^, 10.59 × 10^−2^; *P*-trend = 0.016) compared with the lowest tertile. Similarly, each 1 mg/d higher betaine intake was associated with more favorable changes in executive function (*β* = 7.48 × 10^−4^; 95% CI: 1.71 × 10^−4^, 13.20 × 10^−4^; *P* = 0.011) and language (*β* = 9.13 × 10^−4^; 95% CI: 2.96 × 10^−4^, 15.31 × 10^−4^; *P* = 0.004). Participants in the highest betaine tertile also exhibited greater 2-y improvements in language (*β* = 4.71 × 10^−2^; 95% CI: 0.25 × 10^−2^, 9.17 × 10^−2^; *P*-trend = 0.036) compared with the lowest tertile. All results remained significant after removal of participants with baseline MMSE <24 in both cases (Supplemental Tables 6 and 7).

**TABLE 3:** Longitudinal association between energy-adjusted cumulative average dietary choline intake and changes in cognitive function over 2 y of follow-up in the PREDIMED-Plus cohort

Characteristic	Continuous		Categories of dietary choline intake (mg/d)			*P*-trend
	Dietary choline intake (mg/d)		1st tertile	2nd tertile	3rd tertile	
	*β* (95% CI)^1^	*P* value	*β* (95% CI)	*β* (95% CI)^2^	*β* (95% CI)^2^	
Global Cognitive Function (*n*)	(*n* = 4557)	—	(*n* = 1519)	(*n* = 1519)	(*n* = 1519)	—
Mean ± SD dietary choline intake	416.8 ± 62.7	—	349.9 ± 32.0	415.9 ± 15.0	484.8 ± 37.9	—
Basic model	1.39 (−1.24, 4.03)	0.300	Reference	0.97 (−2.85, 4.81)	1.35 (−2.63, 5.35)	0.506
Multivariable-adjusted model	1.82 (−0.93, 4.57)	0.196	Reference	1.35 (−2.52, 5.22)	1.70 (−2.40, 5.79)	0.422
General Cognitive Function (*n*)	(*n* = 5488)	—	(*n* = 1830)	(*n* = 1829)	(*n* = 1829)	—
Mean ± SD dietary choline intake	418.7 ± 63.4	—	350.9 ± 32.1	417.6 ± 15.2	487.6 ± 38.0	—
Basic model	0.19 (−3.37, 3.75)	0.917	Reference	0.69 (−4.46, 5.85)	−1.70 (−7.14, 3.74)	0.531
Multivariable-adjusted model	−0.42 (−4.11, 3.26)	0.820	Reference	0.14 (−5.11, 5.39)	−2.92 (−8.50, 2.67)	0.294
Executive Function (*n*)	(*n* = 4720)	—	(*n* = 1574)	(*n* = 1573)	(*n* = 1573)	—
Mean ± SD dietary choline intake	417.7 ± 63.4	—	350.3 ± 32.1	416.6 ± 14.9	486.3 ± 39.5	—
Basic model	2.00 (−0.76, 4.76)	0.156	Reference	2.56 (−1.47, 6.61)	3.18 (−0.98, 7.34)	0.136
Multivariable-adjusted model	1.84 (−1.03, 4.72)	0.209	Reference	2.44 (−1.58, 6.47)	2.71 (−1.55, 6.98)	0.220
Attention (*n*)	(*n* = 4797)	—	(*n* = 1599)	(*n* = 1599)	(*n* = 1599)	—
Mean ± SD dietary choline intake	417.7 ± 63.6	—	350.1 ± 32.3	416.6 ± 14.9	486.4 ± 39.8	—
Basic model	5.56 (2.11, 9.01)	0.002^3^	Reference	2.45 (−2.59, 7.51)	8.44 (3.21, 13.71)	0.002^3^
Multivariable-adjusted model	5.20 (1.61, 8.79)	0.005^3^	Reference	1.73 (−3.43, 6.90)	7.50 (2.12, 12.88)	0.006^3^
Language (*n*)	(*n* = 5651)	—	(*n* = 1884)	(*n* = 1884)	(*n* = 1883)	—
Mean ± SD dietary choline intake	419.4 ± 63.9	—	351.3 ± 32.3	418.2 ± 15.2	488.7 ± 39.2	—
Basic model	5.05 (2.03, 8.07)	0.001^3^	Reference	2.87 (−1.48, 7.22)	7.83 (3.22, 12.53)	0.001^3^
Multivariable-adjusted model	3.79 (0.62, 6.95)	0.019^3^	Reference	2.02 (−2.33, 6.38)	5.82 (1.04, 10.59)	0.016^3^

Basic models were adjusted for respective cognitive test score at baseline, age (y), and sex (male or female). Multivariable-adjusted models were further adjusted for intervention group (control or intervention), recruiting center size (<200, 200–300, 300–450, or >450), education level (primary, secondary, or college), marital status (single, divorced, or separated, married, or widower), BMI (kg/m^2^), physical activity (metabolic equivalents in min/d), smoking status (current, former, or never), cumulative average of alcohol consumption in g/d (and adding the quadratic term), depressive symptomatology (yes or no), diabetes prevalence (yes or no), hypertension prevalence (yes or no), hypercholesterolemia prevalence (yes or no), and cumulative average energy-restricted Mediterranean Adherence Screener (low, median, or high). *β*-coefficients (95% CI) were estimated using linear regression models with robust SEs to account for intracluster correlations. Linear trend was calculated by assigning the median values to each tertile of energy-adjusted cumulative average dietary choline intake and treating these values across groups as a continuous variable in the linear regression models.

Abbreviation: CI, confidence interval.

1 *β* (95% CI) values are expressed as multiples of 10^−4^ (×10^−4^).

2 *β* (95% CI) values are expressed as multiples of 10^−2^ (×10^−2^).

3 Indicates significant values (*P* < 0.05).

**TABLE 4:** Longitudinal association between energy-adjusted cumulative average dietary betaine intake and changes in cognitive function over 2 y of follow-up in the PREDIMED-Plus cohort

Characteristic	Continuous		Categories of dietary betaine intake (mg/d)			*P*-trend
	Dietary betaine intake (mg/d)		1st tertile	2nd tertile	3rd tertile	
	*β* (95% CI)^1^	*P* value	*β* (95% CI)	*β* (95% CI)^2^	*β* (95% CI)^2^	
Global Cognitive Function (*n*)	(*n* = 4557)	—	(*n* = 1519)	(*n* = 1519)	(*n* = 1519)	—
Mean ± SD dietary betaine intake	111.6 ± 29.7	—	80.9 ± 13.4	109.9 ± 7.0	144.1 ± 20.4	—
Basic model	−1.92 (−7.06, 3.21)	0.463	Reference	−2.12 (−5.92, 1.67)	−0.69 (−4.55, 3.16)	0.752
Multivariable-adjusted model	2.24 (−3.00, 7.49)	0.402	Reference	−0.52 (−4.29, 3.26)	2.22 (−1.65, 6.09)	0.245
General Cognitive Function (*n*)	(*n* = 5488)	—	(*n* = 1830)	(*n* = 1829)	(*n* = 1829)	—
Mean ± SD dietary betaine intake	112.2 ± 29.6	—	81.5 ± 13.5	110.6 ± 6.90	144.4 ± 20.0	—
Basic model	−5.10 (−12.45, 2.23)	0.173	Reference	−4.72 (−9.84, 0.40)	−1.78 (−6.92, 3.36)	0.534
Multivariable-adjusted model	−2.08 (−9.56, 5.41)	0.587	Reference	−3.54 (−8.70, 1.61)	0.36 (−4.86, 5.58)	0.832
Executive Function (*n*)	(*n* = 4720)	—	(*n* = 1574)	(*n* = 1573)	(*n* = 1573)	—
Mean ± SD dietary betaine intake	111.8 ± 29.8	—	81.0 ± 13.5	110.2 ± 6.9	144.4 ± 20.5	—
Basic model	2.11 (−3.47, 7.70)	0.458	Reference	0.52 (−3.56, 4.59)	0.01 (−4.10, 4.12)	0.996
Multivariable-adjusted model	7.48 (1.71, 13.20)	0.011^3^	Reference	2.43 (−1.64, 6.52)	3.88 (−0.25, 8.02)	0.068
Attention (*n*)	(*n* = 4797)	—	(*n* = 1599)	(*n* = 1599)	(*n* = 1599)	—
Mean ± SD dietary betaine intake	111.9 ± 29.8	—	81.1 ± 13.5	110.3 ± 6.9	144.4 ± 20.7	—
Basic model	−8.97 (−15.75, −2.19)	0.009^3^	Reference	−5.27 (−10.16, −0.38)	−3.86 (−8.92, 1.19)	0.148
Multivariable-adjusted model	−6.07 (−12.92, 0.78)	0.083	Reference	−3.96 (−8.85, 0.93)	−1.64 (−6.74, 3.46)	0.568
Language (*n*)	(*n* = 5651)	—	(*n* = 1884)	(*n* = 1884)	(*n* = 1883)	—
Mean ± SD dietary betaine intake	112.3 ± 29.5	—	81.6 ± 13.5	110.8 ± 6.9	144.6 ± 20.0	—
Basic model	4.51 (−1.54, 10.55)	0.144	Reference	−0.47 (−4.95, 4.01)	1.34 (−3.09, 5.77)	0.541
Multivariable-adjusted model	9.13 (2.96, 15.31)	0.004^3^	Reference	1.28 (−3.19, 5.75)	4.71 (0.25, 9.17)	0.036^3^

Basic models were adjusted for respective cognitive test score at baseline, age (y), and sex (male or female). Multivariable-adjusted models were further adjusted for intervention group (control or intervention), recruiting center size (<200, 200–300, 300–450, or >450), education level (primary, secondary, or college), marital status (single, divorced, or separated, married, or widower), BMI (kg/m^2^), physical activity (metabolic equivalents in min/d), smoking status (current, former, or never), cumulative average of alcohol consumption in g/d (and adding the quadratic term), depressive symptomatology (yes or no), diabetes prevalence (yes or no), hypertension prevalence (yes or no), hypercholesterolemia prevalence (yes or no), and cumulative average energy-restricted Mediterranean Adherence Screener (low, median, or high). *β*-coefficients (95% CI) were estimated using linear regression models with robust SEs to account for intracluster correlations. Linear trend was calculated by assigning the median values to each tertile of energy-adjusted cumulative average dietary choline intake and treating these values across groups as a continuous variable in the linear regression models.

Abbreviation: CI, confidence interval.

1 *β* (95% CI) values are expressed as multiples of 10^−4^ (×10^−4^).

2 *β* (95% CI) values are expressed as multiples of 10^−2^ (×10^−2^).

3 Indicates significant values (*P* < 0.05).

Supplemental Figures 1 and 2 illustrate the results of the post hoc interaction observed between energy-adjusted cumulative average dietary choline and betaine intake and various baseline variables potentially related to cognitive function. No significant interactions were found for any cognitive performance composite score after BH correction (all *q* ≥ 0.050)**.**

## Discussion

This study contributes to the limited body of research investigating the longitudinal relationship between dietary betaine and choline intake and cognitive function. It provides significant and novel findings over time, especially for dietary choline intake, evidenced by the positive dose–response relationship with lower attention decline and higher language domains, even after adjusting for several potential confounders. In parallel, dietary betaine intake was modestly associated with executive function and showed a dose–response relationship with language, with no significant associations observed in other cognitive domains in a population at high risk for cognitive decline. Taken together, these results underscore the potential role of dietary choline and betaine intake on mitigating cognitive decline in older individuals vulnerable to cardiovascular diseases and cognitive impairment.

Longitudinal studies have assessed the association between dietary choline intake and cognitive performance to date relying only on single tests rather than composite domain scores reporting modest magnitudes that are comparable with the small effect sizes observed in our study and are generally considered subtle within the context of cognitive aging research [[14], [15], [16]]. In this line, in a 22-y prospective study of Chinese adults aged 55–79, higher dietary choline intake was associated with modest changes in cognitive function, particularly in memory tests [16]. Similarly, in a United States cohort of adults aged 36–83, higher concurrent dietary choline intake was associated with modest better scores in tests evaluating verbal and visual memory trajectories, although not with verbal learning or executive function [14]. These findings align with those found in a Finnish cohort of males aged 42–60, in which higher dietary choline intake predicted modest differences in memory performance tests scores 4 y thereafter [15]. Collectively, these results suggest that choline may play a specific role in preserving memory-related neural pathways.

The cognitive beneficial effects of betaine have mainly been studied in small supplementation trials showing neuroprotective benefits [46], although the high doses used exceed typical dietary intake, limiting comparability [47]. Observational data on dietary betaine and human cognition remain inconclusive. Despite a consistent association between dietary betaine and better, but modest trajectories of verbal episodic memory among females throughout the menopause transition, no significant associations with other cognitive domains, especially among premenopausal females were observed [8]. Moreover, maternal dietary betaine intake showed no relationship with improved cognitive and language domains of the Bayley test in their children [23]. Taken together, despite the observed modest beneficial associations between dietary choline and betaine intake and cognitive performance in our study, this research question remains unclear to date, particularly in younger, well-nourished populations where cognitive impairment may still be in a preclinical stage. Therefore, further human studies are needed to clarify their role in cognitive function [14].

Interpretation of short-term cognitive change should consider *1*) practice/learning effects, *2*) test–retest variability, and *3*) the test–retest reliability of the instruments. Because all participants completed the same cognitive battery under identical procedures and timing, any generic practice/learning effects are expected to be largely nondifferential across exposure tertiles and therefore unlikely to account for the observed between-tertile differences in 2-y change, which are central to our inference [48]. Nonetheless, the overall mean improvements over follow-up should be interpreted cautiously and not as clinically meaningful cognitive gains at the individual level. Practice effects generally attenuate as retest intervals lengthen, although they may not fully disappear and can differ by test and participant characteristics [39]. Thus, the single reassessment after 2 y likely reduced, but did not eliminate, retesting-related gains, supporting our emphasis on between-tertile comparisons rather than absolute mean changes [49]. Importantly, the magnitude of the observed associations is comparable with that reported in other longitudinal studies, in which short-term cognitive changes among cognitively unimpaired older adults are typically modest [48,50]. Given that retest effects tend to diminish with longer intervals and fewer administrations, the single follow-up after a relatively long 2-y period in this cohort likely further limited practice effects [51]. Overall, because the observed changes may fall within expected test–retest variability, they should be interpreted as subtle shifts in cognitive trajectories that may still be relevant at the population level, particularly in groups at elevated cardiometabolic risk. This interpretation is consistent with evidence that aging can produce domain-specific declines alongside stability or modest improvements in some aspects of attention and executive function through experience and compensatory mechanisms [52].

Adequate dietary choline intake is important for cognitive function during aging, with recommended adequate intakes of 550 mg/d for males and 425 mg/d for females [53]. However, usual intake levels may not capture choline’s full cognitive potential beyond memory [14]. In contrast, typical dietary betaine intake may be insufficient to confer cognitive benefits in older adults [54], although betaine can partially spare choline requirements, with ≤50% of choline needs potentially substituted by betaine [55]. Given that participants’ choline intake was close to the adequate intake over time, this may partly explain why choline, rather than betaine, was more closely associated with cognitive trajectories during follow-up.

Several hypotheses have been proposed to explain the potential relationship between dietary choline and betaine intake and cognition. Choline may support cognitive function through its role as a precursor of acetylcholine and phosphatidylcholine [56], and via betaine-mediated methylation pathways involved in homocysteine remethylation and S-adenosyl-methionine formation [16]. Higher choline intake may increase Trimethylamine N-oxide (TMAO), a gut microbiota–derived metabolite potentially linked to cognitive decline, although its neurodegenerative role remains unclear and microbiota-dependent; thus, choline may be neuroprotective yet carry potential metabolic risks [57]. In contrast, betaine may confer neuroprotection through osmoprotective, methyl-donor, antioxidant, anti-inflammatory, and mitochondrial-supporting actions [58]. Given the chronic inflammation and vascular dysregulation characteristic of metabolic syndrome, these cognitive associations may be amplified in metabolically vulnerable individuals, particularly among those with higher adherence to the Mediterranean diet [59].

This study has several strengths. Its longitudinal design enabled the evaluation of temporal associations over a 2-y follow-up, and cognitive function was comprehensively assessed using multiple neuropsychological tests to derive composite domain scores. The large sample size allowed adjustment for multiple confounders, and findings were supported by sensitivity analyses. However, limitations should be acknowledged. Residual confounding and reverse causality cannot be excluded, particularly from unmeasured factors such as socioeconomic status or family history of dementia. Generalizability may be limited to older adults with overweight or obesity and metabolic syndrome. Although analyses were adjusted for intervention group, the randomized trial context and lifestyle counseling may have influenced cognitive outcomes [29]. Of note, dietary patterns and physical activity have a direct impact on body weight which is also critical, as overweight or obesity has been linked to cognitive decline [60]. The lack of direct measures of cognitive reserve and dynamic dietary adherence should be considered when interpreting heterogeneity in cognitive responses. Although analyses were adjusted for intervention group and cumulative adherence to the energy-restricted Mediterranean diet, both arms followed a Mediterranean pattern, which may partly explain the association between choline intake and overall diet quality. Although erMEDAS captures a global dietary pattern and choline a specific nutrient with substantial variability, residual confounding by correlated dietary components cannot be excluded. Moreover, repeated neuropsychological assessments may have introduced practice effects, which were mitigated but not eliminated through baseline anchoring and adjustment. In addition, participants were cognitively unimpaired at baseline by design, and the trial context, including diet and lifestyle counseling, may have contributed to short-term changes, warranting cautious interpretation of average improvements. Finally, despite using energy-adjusted cumulative intake to better capture long-term exposure, dietary choline and betaine were estimated using FFQs, which may introduce nondifferential measurement error and recall bias [61]. In addition, nutrient values were derived from USDA food composition tables because Spanish databases lack comprehensive and systematically updated data, although differences in food composition or preparation methods may introduce some degree of nutrient misclassification, this error is expected to be largely nondifferential [59].

In conclusion, our results suggest that higher daily dietary choline and betaine intake over time may be associated with modest favorable changes in cognitive function and may help to mitigate cognitive decline at short term in older adults with overweight/obesity and metabolic syndrome. Further research in this area aimed at increasing dietary choline and betaine intake in individuals at risk of cognitive impairment over a longer follow-up period is warranted, given the rapidly expanding elderly population and the absence of curative treatment for cognitive decline.

## Patient consent

All participants signed an informed consent form upon entry into the study.

## Author contributions

The authors’ responsibilities were as follows – HV-L: writing – original draft, writing – review and editing, methodology, investigation, data curation, conceptualization; JMM-E: writing – original draft, writing – review and editing, methodology, formal analysis, data curation, conceptualization; NB: writing – review and editing, investigation, data curation, conceptualization, funding acquisition; MR-C: writing – review and editing, funding acquisition; DC: writing – review and editing, funding acquisition; JH: writing – review and editing, funding acquisition; JAM: writing – review and editing, funding acquisition; ÁMA-G: writing – review and editing, funding acquisition; JW: writing – review and editing, funding acquisition; JV: writing – review and editing, funding acquisition; DR: writing – review and editing, funding acquisition; JL-M: writing – review and editing, funding acquisition; RE: writing – review and editing, funding acquisition; FJT: writing – review and editing, funding acquisition; VU-F: writing – review and editing, funding acquisition; LS-M: writing – review and editing, funding acquisition; NC-I: writing – review and editing, funding acquisition; JAT: writing – review and editing, funding acquisition; VMS: writing – review and editing, funding acquisition; XP: writing – review and editing, funding acquisition; MD-R: writing – review and editing, funding acquisition; PM-M: writing – review and editing, funding acquisition; JV: writing – review and editing, funding acquisition; CV: writing – review and editing, funding acquisition; ER: writing – review and editing, funding acquisition; FF-A: writing – review and editing; ET: writing – review and editing; LG-C: writing – review and editing; JVS: writing – review and editing; MDZ: writing – review and editing; AG-R: writing – review and editing; AO-C: writing – review and editing; RC-G: writing – review and editing; MAZ: writing – review and editing; LP: writing – review and editing; RC: writing – review and editing; MD-L: writing – review and editing; LT-S: writing – review and editing; VS-F: writing – review and editing; ZV-R: writing – review and editing; RF-C: writing – review and editing; OC: writing – review and editing; ALR-M: writing – review and editing; AA: writing – review and editing; AG-A: writing – review and editing; MF: writing – review and editing; LD: writing – review and editing, investigation, data curation, conceptualization, funding acquisition; JS-S: writing – original draft, writing – review and editing, visualization, validation, methodology, investigation, data curation, conceptualization, project administration, funding acquisition; and all authors: read and approved the final manuscript.

## Ethics approval

The study protocol was approved by the Research Ethics Committees of all recruiting centers.

## Data availability

Data described in the manuscript, codebook, and analytic code will be made available upon request pending application and approval of the PREDIMED-Plus Steering Committee. There are restrictions on the availability of data for the PREDIMED-Plus trial, because of the signed consent agreements around data sharing, which only allow access to external researchers for studies following the project purposes. Requestors wishing to access the PREDIMED-Plus trial data used in this study can make a request to the PREDIMED-Plus trial Steering Committee chair: jordi.salas@urv.cat. The request will then be passed to members of the PREDIMED-Plus Steering Committee for deliberation.

## Declaration of generative AI and AI-assisted technologies in the writing process

During the preparation of this work the authors used no tool or service.

## Funding

This study was supported by the official Spanish Institutions for Funding Scientific Biomedical Research, CIBER Fisiopatología de la Obesidad y Nutrición (CIBEROBN), and Instituto de Salud Carlos III (ISCIII), through the Fondo de Investigación para la Salud (FIS), which is cofunded by the European Regional Development Fund (6 coordinated FIS projects led by JSS and JV, including the following projects: PI13/00673, PI13/00492, PI13/00272, PI13/01123, PI13/00462, PI13/00233, PI13/02184, PI13/00728, PI13/01090, PI13/01056, PI14/01722, PI14/00636, PI14/00618, PI14/00696, PI14/01206, PI14/01919, PI14/00853, PI14/01374, PI14/00972, PI14/00728, PI14/01471, PI16/00473, PI16/00662, PI16/01873, PI16/01094, PI16/00501, PI16/00533, PI16/00381, PI16/00366, PI16/01522, PI16/01120, PI17/00764, PI17/01183, PI17/00855, PI17/01347, PI17/00525, PI17/01827, PI17/00532, PI17/00215, PI17/01441, PI17/00508, PI17/01732, PI17/00926, PI19/00957, PI19/00386, PI19/00309, PI19/01032, PI19/00576, PI19/00017, PI19/01226, PI19/00781, PI19/01560, PI19/01332, PI20/01802, PI20/00138, PI20/01532, PI20/00456, PI20/00339, PI20/00557, PI20/00886, and PI20/01158); the Especial Action Project entitled: Implementación y evaluación de una intervención intensiva sobre la actividad física Cohorte PREDIMED-Plus grant to JSS; the European Research Council (Advanced Research Grant 2014–2019; agreement #340918) granted to MMG; the Recercaixa (number 2013ACUP00194) grant to JSS; grants from the Consejería de Salud de la Junta de Andalucía (PI0458/2013, PS0358/2016, and PI0137/2018); the PROMETEO/21/2021 and the AICO/2021/347 grants from the Generalitat Valenciana; and the Horizon 2020 PRIME study (Prevention and Remediation of Insulin Multimorbidity in Europe; grant agreement #847879). HVL holds a Sara Borrell (CD25/00181) research contract from Instituto de Salud Carlos III, cofounded by the European Social Fund (ESF). None of the funding sources took part in the design, collection, analysis, interpretation of the data, or writing the report, or in the decision to submit the manuscript for publication. JSS, senior author, gratefully acknowledges the financial support by ICREA under the ICREA Academia program.

## Conflict of interest

The authors declare that there are no known financial interests or personal relationships that could have appeared to influence the work reported in this manuscript.
