# Supplementary material for: Dietary Choline and Betaine Intake and 2-year Changes in Cognitive Function in Older Adults With Overweight or Obesity and Metabolic Syndrome: A Prospective Cohort Analysis
Source: Am J Clin Nutr. 2026 Mar 11;123(5):101265. doi: 10.1016/j.ajcnut.2026.101265 (PMC13197923; doi:10.1016/j.ajcnut.2026.101265)
Supplement: multimedia component 1 [file mmc1.docx]

| **Table S1.-** Composite cognitive assessment equations^1^ | |
| --- | --- |
| **Composite cognitive domain** | **Composite component score** |
| *Global Cognitive Function^2^* | = $\frac{zMMSE + zCDT+ zVFT-a + zVFT-p + \left( -zTMT-A \right)+ \left( -zTMT-B \right)+ zDST-f+ zDST-b}{8}$ |
| *General Cognitive Function^3^* | = $\frac{zMMSE + zCDT}{2}$ |
| *Executive Function^4^* | = $\frac{zVFT-a + zVFT-p + \left( -zTMT-B \right) + zDST-b}{4}$ |
| *Attention^5^* | = $\frac{\left( -zTMT-A \right)+ zDST-f}{2}$ |
| *Language^6^* | = $\frac{zVFT-a + zVFT-p}{2}$ |
| Abbreviations: CDT, Clock Drawing Test; DST-b, Digit Span test - backward; DST-f, Digit Span test - forward; MMSE, Mini-Mental State Examination; TMT-A, Trail Making Test Part A; TMT-B, Trail Making Test Part B; VFT-a, Verbal Fluency tasks semantical; VFT-p, Verbal Fluency tasks phonological.  ^1^Standardized scores of the TMT-A and TMT-B were inverted, so that higher scores would represent better cognitive function.  ^2^The global cognitive function was determined by computing the mean standardized individual scores of all neuropsychological tests.  ^3^The general cognitive function composite included the MMSE score and the CDT score.  ^4^The executive function composite included the mean standardized individual scores of the VFT-a score, VFT-p score, TMT-B score, and DST-b score.  ^5^The attention composite included the mean standardized individual scores of the TMT-A score and the DST-f score.  ^6^ The language composite included the mean standardized individual scores of the VFT-a score and the VFT-p score. | |

| **Table S2.-** Changes in cognitive function composite scores over 2 years of follow-up in overall and by tertiles of energy-adjusted cumulative average dietary choline intake in the PREDIMED-Plus cohort. | | | | |
| --- | --- | --- | --- | --- |
| **Characteristic** | **All** | **Categories of dietary choline intake** | | |
|  |  | **1^st^ tertile** | **2^nd^ tertile** | **3^rd^ tertile** |
| **Δ Global Cognitive Function (n)** | (n = 4557) | (n = 1519) | (n = 1519) | (n = 1519) |
| Mean ± SD dietary choline intake | 416.8 ± 62.7 | 349.9 ± 32.0 | 415.9 ± 15.0 | 484.8 ± 37.9 |
| Mean Δ [min to max] | 0.04 [–3.31, 2.84] | 0.05 [–3.31, 2.84] | 0.03 [–3.24, 2.47] | 0.03 [–2.12, 2.66] |
| **Δ General Cognitive Function (n)** | (n = 5488) | (n = 1830) | (n = 1829) | (n = 1829) |
| Mean ± SD dietary choline intake | 418.7 ± 63.4 | 350.9 ± 32.1 | 417.6 ± 15.2 | 487.6 ± 38.0 |
| Mean Δ [min to max] | 0.09 [–3.75, 5.46] | 0.13 [–3.75, 4.42] | 0.09 [–3.57, 4.43] | 0.06 [–3.75, 5.46] |
| **Δ Executive Function (n)** | (n = 4720) | (n = 1574) | (n = 1573) | (n = 1573) |
| Mean ± SD dietary choline intake | 417.7 ± 63.4 | 350.3 ± 32.1 | 416.6 ± 14.9 | 486.3 ± 39.5 |
| Mean Δ [min to max] | 0.04 [–2.49, 2.78] | 0.04 [–2.46, 2.78] | 0.05 [–2.49, 2.25] | 0.03 [–2.48, 2.59] |
| **Δ Attention** **(n)** | (n = 4797) | (n = 1599) | (n = 1599) | (n = 1599) |
| Mean ± SD dietary choline intake | 417.7 ± 63.6 | 350.1 ± 32.3 | 416.6 ± 14.9 | 486.4 ± 39.8 |
| Mean Δ [min to max] | –0.04 [–6.06, 6.03] | –0.06 [–6.06, 3.76] | –0.06 [–5.38, 5.52] | –0.01 [–5.21, 6.02] |
| **Δ Language (n)** | (n = 5651) | (n = 1884) | (n = 1884) | (n = 1883) |
| Mean ± SD dietary choline intake | 419.4 ± 63.9 | 351.3 ± 32.3 | 418.2 ± 15.2 | 488.7 ± 39.2 |
| Mean Δ [min to max] | 0.11 [–3.69, 6.27] | 0.09 [–3.35, 3.18] | 0.10 [–3.20, 2.92] | 0.13 [–3.70, 6.28] |
| Abbreviations: max, maximum; min, minimum; SD, standard deviation.  Data are presented as mean ± SD energy-adjusted cumulative average dietary choline intake, and changes (Δ) [min to max] in cognitive function variables, respectively. | | | | |

| **Table S3.-** Changes in cognitive function composite scores over 2 years of follow-up in overall and by tertiles of energy-adjusted cumulative average dietary betaine intake in the PREDIMED-Plus cohort. | | | | |
| --- | --- | --- | --- | --- |
| **Characteristic** | **All** | **Categories of dietary betaine intake** | | |
|  |  | **1^st^ tertile** | **2^nd^ tertile** | **3^rd^ tertile** |
| **Δ Global Cognitive Function (n)** | (n = 4557) | (n = 1519) | (n = 1519) | (n = 1519) |
| Mean ± SD dietary choline intake | 111.6 ± 29.7 | 80.9 ± 13.4 | 109.9 ± 7.0 | 144.1 ± 20.4 |
| Mean Δ [min to max] | 0.04 [–3.31, 2.84] | 0.04 [–2.07, 2.05] | 0.03 [–3.31, 2.84] | 0.04 [–2.98, 2.66] |
| **Δ General Cognitive Function (n)** | (n = 5488) | (n = 1830) | (n = 1829) | (n = 1829) |
| Mean ± SD dietary choline intake | 112.2 ± 29.6 | 81.5 ± 13.5 | 110.6 ± 6.90 | 144.4 ± 20.0 |
| Mean Δ [min to max] | 0.09 [–3.75, 5.46] | 0.09 [–3.63, 4.43] | 0.08 [–3.75, 5.46] | 0.12 [–3.76, 4.64] |
| **Δ Executive Function (n)** | (n = 4720) | (n = 1574) | (n = 1573) | (n = 1573) |
| Mean ± SD dietary choline intake | 111.8 ± 29.8 | 81.0 ± 13.5 | 110.2 ± 6.9 | 144.4 ± 20.5 |
| Mean Δ [min to max] | 0.04 [–2.49, 2.78] | 0.03 [–2.16, 1.99] | 0.04 [–2.47, 2.78] | 0.04 [–2.49, 2.67] |
| **Δ Attention** **(n)** | (n = 4797) | (n = 1599) | (n = 1599) | (n = 1599) |
| Mean ± SD dietary choline intake | 111.9 ± 29.8 | 81.1 ± 13.5 | 110.3 ± 6.9 | 144.4 ± 20.7 |
| Mean Δ [min to max] | –0.04 [–6.06, 6.03] | –0.02 [–4.58, 5.11] | –0.06 [–5.42, 6.02] | –0.04 [–6.06, 6.02] |
| **Δ Language (n)** | (n = 5651) | (n = 1884) | (n = 1884) | (n = 1883) |
| Mean ± SD dietary choline intake | 112.3 ± 29.5 | 81.6 ± 13.5 | 110.8 ± 6.9 | 144.6 ± 20.0 |
| Mean Δ [min to max] | 0.11 [–3.69, 6.27] | 0.09 [–2.86, 6.28] | 0.10 [–3.70, 4.23] | 0.12 [–3.35, 3.18] |
| Abbreviations: max, maximum; min, minimum; SD, standard deviation.  Data are presented as mean ± SD energy-adjusted cumulative average dietary betaine intake, and changes (Δ) [min to max] in cognitive function variables, respectively. | | | | |

| **Table S4.-** Changes in cognitive function individual tests over 2 years of follow-up in overall and by tertiles of energy-adjusted cumulative average dietary choline intake in the PREDIMED-Plus cohort. | | | | |
| --- | --- | --- | --- | --- |
| **Characteristic** | **All** | **Categories of dietary choline intake** | | |
|  |  | **1^st^ tertile** | **2^nd^ tertile** | **3^rd^ tertile** |
| **Δ MMSE (n)** | (n = 5504) | (n = 1835) | (n = 1835) | (n = 1834) |
| Mean ± SD dietary choline intake | 418.7 ± 63.3 | 350.9 ± 32.1 | 417.6 ± 15.2 | 487.5 ± 38.0 |
| Mean Δ [min to max] | 0.11 [–4.28, 5.35] | 0.11 [–3.74, 4.28] | 0.14 [–4.28, 5.35] | 0.05 [–3.74, 3.74] |
| **Δ CDT (n)** | (n = 5505) | (n = 1835) | (n = 1835) | (n = 1835) |
| Mean ± SD dietary choline intake | 418.7 ± 63.4 | 350.9 ± 32.1 | 417.6 ± 15.2 | 487.8 ± 38.1 |
| Mean Δ [min to max] | 0.04 [–5.78, 5.78] | 0.10 [–4.13, 4.96] | 0.01 [–4.13, 4.96] | 0.01 [–5.78, 5.78] |
| **Δ VFT-a (n)** | (n = 5653) | (n = 1885) | (n = 1884) | (n = 1884) |
| Mean ± SD dietary choline intake | 419.4 ± 63.9 | 351.2 ± 32.4 | 418.2 ± 15.2 | 488.7 ± 39.2 |
| Mean Δ [min to max] | 0.10 [–4.10, 5.32] | 0.10 [–3.89, 3.48] | 0.10 [–3.89, 4.51] | 0.09 [–4.09, 5.32] |
| **Δ VFT-p** **(n)** | (n = 5652) | (n = 1884) | (n = 1884) | (n = 1884) |
| Mean ± SD dietary choline intake | 419.4 ± 63.9 | 351.2 ± 32.3 | 418.2 ± 15.2 | 487.7 ± 39.2 |
| Mean Δ [min to max] | 0.08 [–4.45, 5.78] | 0.05 [–4.45, 3.78] | 0.07 [–3.56, 3.34] | 0.13 [–3.11, 5.78] |
| **Δ TMT-A (n)** | (n = 5603) | (n = 1868) | (n = 1868) | (n = 1867) |
| Mean ± SD dietary choline intake | 419.3 ± 64.0 | 351.2 ± 32.4 | 418.0 ± 15.2 | 488.6 ± 39.4 |
| Mean Δ [min to max] | 0.04 [–8.70, 8.91] | 0.06 [–5.53, 8.77] | 0.06 [–8.09, 8.91] | –0.01 [–8.70, 7.01] |
| **Δ TMT-B (n)** | (n = 5506) | (n = 1836) | (n = 1835) | (n = 1835) |
| Mean ± SD dietary choline intake | 419.3 ± 63.7 | 351.5 ± 32.1 | 418.1 ± 15.2 | 488.6 ± 38.9 |
| Mean Δ [min to max] | 0.03 [–3.88, 3.50] | 0.01 [–3.70, 3.41] | 0.05 [–3.88, 3.50] | 0.03 [–3.52, 3.50] |
| **Δ DST-f (n)** | (n = 4841) | (n = 1614) | (n = 1614) | (n = 1613) |
| Mean ± SD dietary choline intake | 417.7 ± 63.6 | 350.0 ± 32.3 | 416.7 ± 15.0 | 486.5 ± 39.7 |
| Mean Δ [min to max] | –0.03 [–5.70, 4.08] | –0.03 [–4.07, 3.26] | –0.04 [–5.70, 4.07] | –0.02 [–3.67, 3.67] |
| **Δ DST-b (n)** | (n = 4840) | (n = 1614) | (n = 1613) | (n = 1613) |
| Mean ± SD dietary choline intake | 417.7 ± 63.6 | 350.0 ± 32.3 | 416.7 ± 15.0 | 486.5 ± 39.7 |
| Mean Δ [min to max] | –0.01 [–4.55, 4.55] | –0.02 [–4.09, 3.18] | 0.02 [–4.54, 4.54] | –0.02 [–4.09, 4.09] |
| Abbreviations: CDT, Clock Drawing Test; DST-b, Digit Span test - backward; DST-f, Digit Span test - forward; max, maximum; min, minimum; MMSE, Mini-Mental State Examination; SD, standard deviation; TMT-A, Trail Making Test Part A; TMT-B, Trail Making Test Part B; VFT-a, Verbal Fluency tasks semantical; VFT-p, Verbal Fluency tasks phonological.  Data are presented as mean ± SD energy-adjusted cumulative average dietary choline intake, and changes (Δ) [min to max] in cognitive function variables, respectively. | | | | |

| **Table S5.-** Changes in cognitive function individual tests over 2 years of follow-up in overall and by tertiles of energy-adjusted cumulative average dietary betaine intake in the PREDIMED-Plus cohort. | | | | |
| --- | --- | --- | --- | --- |
| **Characteristic** | **All** | **Categories of dietary betaine intake** | | |
|  |  | **1^st^ tertile** | **2^nd^ tertile** | **3^rd^ tertile** |
| **Δ MMSE (n)** | (n = 5504) | (n = 1835) | (n = 1835) | (n = 1834) |
| Mean ± SD dietary choline intake | 112.2 ± 29.5 | 81.6 ± 13.5 | 110.6 ± 6.9 | 144.4 ± 20.0 |
| Mean Δ [min to max] | 0.11 [–4.28, 5.35] | 0.09 [–3.74, 3.74] | 0.06 [–4.28, 5.35] | 0.15 [–3.74, 4.82] |
| **Δ CDT (n)** | (n = 5505) | (n = 1835) | (n = 1835) | (n = 1835) |
| Mean ± SD dietary choline intake | 112.2 ± 29.5 | 81.6 ± 13.5 | 110.6 ± 6.9 | 144.4 ± 20.0 |
| Mean Δ [min to max] | 0.04 [–5.78, 5.78] | 0.06 [–5.78, 4.96] | 0.05 [–4.13, 5.78] | 0.02 [–5.78, 5.78] |
| **Δ VFT-a (n)** | (n = 5653) | (n = 1885) | (n = 1884) | (n = 1884) |
| Mean ± SD dietary choline intake | 112.3 ± 29.5 | 81.6 ± 13.6 | 110.8 ± 6.9 | 124.4 ± 39.3 |
| Mean Δ [min to max] | 0.10 [–4.10, 5.32] | 0.10 [–3.89, 5.32] | 0.10 [–4.10, 4.51] | 0.10 [–3.69, 3.48] |
| **Δ VFT-p** **(n)** | (n = 5652) | (n = 1884) | (n = 1884) | (n = 1884) |
| Mean ± SD dietary choline intake | 112.3 ± 29.5 | 81.6 ± 13.6 | 110.8 ± 6.9 | 124.4 ± 39.3 |
| Mean Δ [min to max] | 0.08 [–4.45, 5.78] | 0.07 [–3.11, 5.78] | 0.07 [–4.45, 4.00] | 0.11 [–2.89, 3.78] |
| **Δ TMT-A (n)** | (n = 5603) | (n = 1868) | (n = 1868) | (n = 1867) |
| Mean ± SD dietary choline intake | 112.3 ± 29.5 | 81.6 ± 13.5 | 110.8 ± 6.9 | 144.6 ± 20.0 |
| Mean Δ [min to max] | 0.04 [–8.70, 8.91] | 0.04 [–8.08, 8.37] | 0.04 [–8.70, 8.81] | 0.03 [–8.30, 8.91] |
| **Δ TMT-B (n)** | (n = 5506) | (n = 1836) | (n = 1835) | (n = 1835) |
| Mean ± SD dietary choline intake | 112.2 ± 29.5 | 81.5 ± 13.5 | 110.6 ± 6.9 | 144.4 ± 19.9 |
| Mean Δ [min to max] | 0.03 [–3.88, 3.50] | 0.04 [–3.88, 3.50] | 0.01 [–3.52, 3.50] | 0.03 [–3.43, 3.25] |
| **Δ DST-f (n)** | (n = 4841) | (n = 1614) | (n = 1614) | (n = 1613) |
| Mean ± SD dietary choline intake | 111.9 ± 29.8 | 81.1 ± 13.5 | 110.3 ± 6.9 | 144.4 ± 20.6 |
| Mean Δ [min to max] | –0.03 [–5.70, 4.08] | –0.01 [–4.07, 3.26] | –0.04 [–4.07, 3.66] | –0.04 [–5.70, 4.07] |
| **Δ DST-b (n)** | (n = 4840) | (n = 1614) | (n = 1613) | (n = 1613) |
| Mean ± SD dietary choline intake | 111.9 ± 29.8 | 81.1 ± 13.5 | 110.3 ± 6.9 | 144.4 ± 20.6 |
| Mean Δ [min to max] | –0.01 [–4.55, 4.55] | 0.01 [–4.54, 4.54] | –0.01 [–3.18, 4.09] | 0.01 [–4.10, 3.63] |
| Abbreviations: CDT, Clock Drawing Test; DST-b, Digit Span test - backward; DST-f, Digit Span test - forward; max, maximum; min, minimum; MMSE, Mini-Mental State Examination; SD, standard deviation; TMT-A, Trail Making Test Part A; TMT-B, Trail Making Test Part B; VFT-a, Verbal Fluency tasks semantical; VFT-p, Verbal Fluency tasks phonological.  Data are presented as mean ± SD energy-adjusted cumulative average dietary betaine intake, and changes (Δ) [min to max] in cognitive function variables, respectively. | | | | |

| **Table S6.-** Sensitivity analysis for the longitudinal association between energy-adjusted cumulative average dietary choline intake and changes in cognitive function over 2 years of follow-up in the PREDIMED-Plus cohort after removal of participants with baseline MMSE <24^1^ | | | | | | |
| --- | --- | --- | --- | --- | --- | --- |
|  | **Continuous** | | **Categories of dietary choline intake (mg per day)** | | |  |
| **Characteristic** | **Dietary choline intake (mg per day)** | | **1^st^ tertile** | **2^nd^ tertile** | **3^rd^ tertile** |  |
|  | **β [95% CI]^2^** | **p-value** | **β [95% CI]** | **β [95% CI]^2^** | **β [95% CI]^3^** | **P-trend** |
| **Global Cognitive Function (n)** | (n = 4449) | **–** | (n = 1483) | (n = 1483) | (n = 1483) | **–** |
| Mean ± SD dietary choline intake | 416.8 ± 62.7 | **–** | 349.9 ± 31.8 | 415.9 ± 15.0 | 484.9 ± 38.2 | **–** |
| Basic model | 1.29 [–1.35, 3.93] | 0.337 | Reference | 1.15 [–2.69, 5.01] | 1.21 [–2.79, 5.22] | 0.553 |
| Multivariable-adjusted model | 1.61 [–1.15, 4.37] | 0.253 | Reference | 1.48 [–2.40, 5.37] | 1.49 [–2.63, 5.60] | 0.487 |
| **General Cognitive Function (n)** | (n = 5329) | **–** | (n = 1777) | (n = 1776) | (n = 1776) | **–** |
| Mean ± SD dietary choline intake | 418.4 ± 63.3 | **–** | 350.8 ± 31.9 | 417.3 ± 15.1 | 487.2 ± 38.2 | **–** |
| Basic model | –0.15 [–3.62, 3.32] | 0.932 | Reference | 0.58 [–4.45, 5.62] | –1.72 [–7.07, 3.63] | 0.521 |
| Multivariable-adjusted model | –1.04 [–4.63, 2.55] | 0.570 | Reference | –0.44 [–5.17, 5.08] | –3.30 [–8.79, 2.19] | 0.229 |
| **Executive Function** **(n)** | (n = 4610) | **–** | (n = 1537) | (n = 1537) | (n = 1536) | **–** |
| Mean ± SD dietary choline intake | 417.7 ± 63.5 | **–** | 350.3 ± 31.9 | 416.5 ± 15.0 | 486.4 ± 39.8 | **–** |
| Basic model | 2.13 [–0.65, 4.92] | 0.134 | Reference | 3.16 [–0.93, 7.25] | 3.22 [–0.99, 7.45] | 0.137 |
| Multivariable-adjusted model | 1.85 [–1.06, 4.75] | 0.212 | Reference | 2.91 [–1.17, 6.99] | 2.63 [–1.71, 6.96] | 0.245 |
| **Attention** **(n)** | (n = 4677) | **–** | (n = 1559) | (n = 1559) | (n = 1559) | **–** |
| Mean ± SD dietary choline intake | 417.7 ± 63.7 | **–** | 350.1 ± 32.1 | 416.5 ± 14.9 | 486.5 ± 40.1 | **–** |
| Basic model | **5.09 [1.63, 8.56]** | **0.004** | Reference | 1.60 [–3.44, 6.64] | **7.65 [2.40, 12.90]** | **0.004** |
| Multivariable-adjusted model | **4.69 [1.09, 8.29]** | **0.011** | Reference | 0.94 [–4.22, 6.11] | **6.74 [1.34, 12.15]** | **0.013** |
| **Language (n)** | (n = 5488) | **–** | (n = 1830) | (n = 1829) | (n = 1829) | **–** |
| Mean ± SD dietary choline intake | 419.2 ± 63.9 | **–** | 351.2 ± 32.1 | 417.9 ± 15.1 | 488.5 ± 39.5 | **–** |
| Basic model | **5.42 [2.33, 8.50]** | **0.001** | Reference | 3.38 [–1.05, 7.82] | **8.26 [3.54, 12.96]** | **0.001** |
| Multivariable-adjusted model | **4.10 [0.87, 7.33]** | **0.013** | Reference | 2.49 [–1.95, 6.94] | **6.09 [1.22, 10.97]** | **0.014** |
| Abbreviations: CI, confidence interval. Basic models were adjusted for respective cognitive test score at baseline, age (years), and sex (male or female). Multivariable-adjusted models were further adjusted for intervention group (control or intervention), recruiting center size (<200, 200-300, 300-450 or >450), education level (primary, secondary, or college), marital status (single, divorced or separated, married, or widower), body mass index (kg/m^2^), physical activity (metabolic equivalents in minutes per day), smoking status (current, former, or never), cumulative average of alcohol consumption in grams per day (and adding the quadratic term), depressive symptomatology (yes or no), diabetes prevalence (yes or no), hypertension prevalence (yes or no), hypercholesterolemia prevalence (yes or no), and cumulative average adherence to energy-restricted Mediterranean diet (low, median, or high). β-coefficients [95% CI] were estimated using linear regression models with robust standard errors to account for intracluster correlations. Linear trend was calculated by assigning the median values to each quartile of energy-adjusted cumulative average dietary choline intake and treating these values across groups as a continuous variable in the linear regression models. Significant values (*p*<0.05) were highlighted in bold type.  ^1^participants with baseline MMSE <24 (n = 203)  ^2^β [95% CI] values are expressed as multiples of 10^-4^ (x10^-4^).  ^3^β [95% CI] values are expressed as multiples of 10^-2^ (x10^-2^). | | | | | | |

| **Table S7.-** Sensitivity analysis for the longitudinal association between energy-adjusted cumulative average dietary betaine intake and changes in cognitive function over 2 years of follow-up in the PREDIMED-Plus cohort after removal of participants with baseline MMSE <24^1^ | | | | | | |
| --- | --- | --- | --- | --- | --- | --- |
|  | **Continuous** | | **Categories of dietary betaine intake (mg per day)** | | |  |
| **Characteristic** | **Dietary betaine intake (mg per day)** | | **1^st^ tertile** | **2^nd^ tertile** | **3^rd^ tertile** |  |
|  | **β [95% CI]^2^** | **p-value** | **β [95% CI]** | **β [95% CI]^2^** | **β [95% CI]^3^** | **P-trend** |
| **Global Cognitive Function (n)** | (n = 4449) | **–** | (n = 1483) | (n = 1483) | (n = 1483) | **–** |
| Mean ± SD dietary betaine intake | 111.4 ± 29.8 | **–** | 80.6 ± 13.4 | 109.7 ± 7.0 | 144.0 ± 20.4 | **–** |
| Basic model | –1.89 [–7.07, 3.29] | 0.475 | Reference | –2.98 [–5.88, 1.71] | –0.56 [–4.45, 3.33] | 0.803 |
| Multivariable-adjusted model | 2.37 [–2.93, 7.67] | 0.380 | Reference | –0.46 [–4.24, 3.31] | 2.40 [–1.49, 6.30] | 0.214 |
| **General Cognitive Function (n)** | (n = 5329) | **–** | (n = 1777) | (n = 1776) | (n = 1776) | **–** |
| Mean ± SD dietary betaine intake | 111.9 ± 29.6 | **–** | 81.2 ± 13.5 | 110.4 ± 6.92 | 144.3 ± 20.0 | **–** |
| Basic model | –4.96 [–12.22, 2.31] | 0.181 | Reference | –4.55 [–9.58, 0.47] | –1.33 [–6.41, 3.76] | 0.647 |
| Multivariable-adjusted model | –1.38 [–8.76, 6.01] | 0.715 | Reference | –3.23 [–8.28, 1.81] | 1.11 [–4.02, 6.24] | 0.617 |
| **Executive Function** **(n)** | (n = 4610) | **–** | (n = 1537) | (n = 1537) | (n = 1536) | **–** |
| Mean ± SD dietary betaine intake | 111.6 ± 29.9 | **–** | 80.7 ± 13.5 | 110.0 ± 6.9 | 144.2 ± 20.5 | **–** |
| Basic model | 2.70 [–2.94, 8.33] | 0.348 | Reference | 0.14 [–3.98, 4.27] | 0.03 [–4.13, 4.21] | 0.989 |
| Multivariable-adjusted model | **8.16 [2.34, 13.97]** | **0.006** | Reference | 2.03 [–2.11, 6.17] | 3.80 [–0.40, 7.99] | 0.077 |
| **Attention** **(n)** | (n = 4677) | **–** | (n = 1559) | (n = 1559) | (n = 1559) | **–** |
| Mean ± SD dietary betaine intake | 111.7 ± 29.9 | **–** | 80.8 ± 13.5 | 110.1 ± 7.0 | 144.3 ± 20.6 | **–** |
| Basic model | **–8.81 [–15.65, –1.97]** | **0.012** | Reference | –4.46 [–9.34, 0.41] | –3.86 [–8.92, 1.19] | 0.180 |
| Multivariable-adjusted model | –6.07 [–13.01, 0.85] | 0.086 | Reference | –3.25 [–8.15, 1.64] | –1.54 [–6.66, 3.59] | 0.588 |
| **Language (n)** | (n = 5488) | **–** | (n = 1830) | (n = 1829) | (n = 1829) | **–** |
| Mean ± SD dietary betaine intake | 112.1 ± 29.6 | **–** | 81.3 ± 13.5 | 110.6 ± 6.9 | 144.4 ± 20.0 | **–** |
| Basic model | 4.87 [–1.25, 10.99] | 0.119 | Reference | –0.47 [–4.95, 4.01] | –3.57 [–8.64, 1.51] | 0.551 |
| Multivariable-adjusted model | **9.41 [3.16, 15.66]** | **0.003** | Reference | 1.27 [–3.29, 5.84] | **4.60 [0.58, 9.14]** | **0.045** |
| Abbreviations: CI, confidence interval. Basic models were adjusted for respective cognitive test score at baseline, age (years), and sex (male or female). Multivariable-adjusted models were further adjusted for intervention group (control or intervention), recruiting center size (<200, 200-300, 300-450 or >450), education level (primary, secondary, or college), marital status (single, divorced or separated, married, or widower), body mass index (kg/m^2^), physical activity (metabolic equivalents in minutes per day), smoking status (current, former, or never), cumulative average of alcohol consumption in grams per day (and adding the quadratic term), depressive symptomatology (yes or no), diabetes prevalence (yes or no), hypertension prevalence (yes or no), hypercholesterolemia prevalence (yes or no), and cumulative average adherence to energy-restricted Mediterranean diet (low, median, or high). β-coefficients [95% CI] were estimated using linear regression models with robust standard errors to account for intracluster correlations. Linear trend was calculated by assigning the median values to each quartile of energy-adjusted cumulative average dietary betaine intake and treating these values across groups as a continuous variable in the linear regression models. Significant values (*p*<0.05) were highlighted in bold type.  ^1^participants with baseline MMSE <24 (n = 203)  ^2^β [95% CI] values are expressed as multiples of 10^-4^ (x10^-4^).  ^3^β [95% CI] values are expressed as multiples of 10^-2^ (x10^-2^). | | | | | | |

**
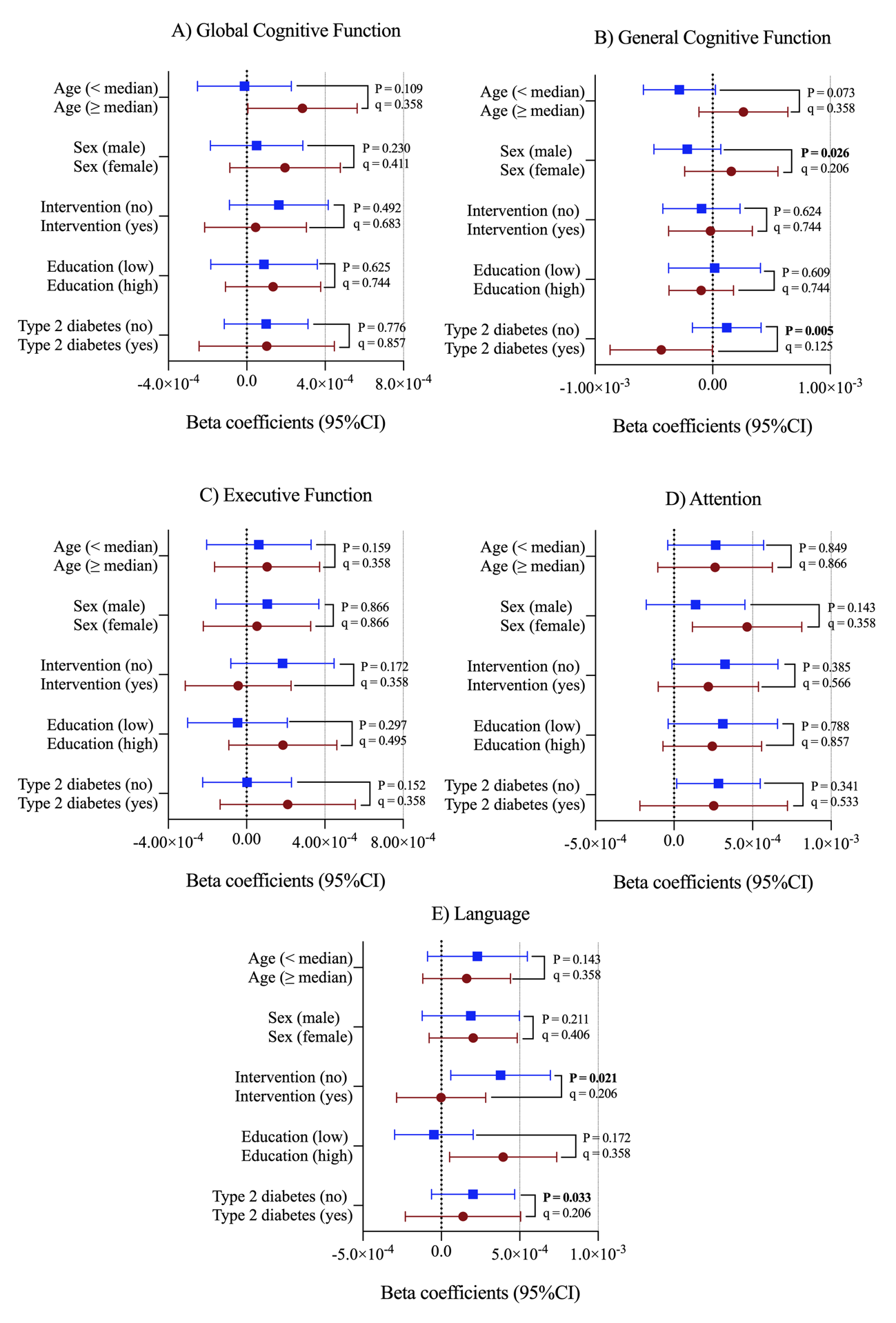
**

**Figure S1.- Interaction between energy-adjusted cumulative average dietary choline intake and different baseline variables of the study potentially related to cognitive function.** Abbreviations: BMI, body mass index; CI, confidence interval. Multivariable-adjusted models were adjusted for respective cognitive test score at baseline, age (years), sex, intervention group (control or intervention), recruiting center size (<200, 200-300, 300-450 or >450), education level (primary, secondary, or college), marital status (single, divorced or separated, married, or widower), body mass index (kg/m^2^), physical activity (metabolic equivalents in minutes per day), smoking status (current, former, or never), cumulative average of alcohol consumption in grams per day (and adding the quadratic term), depressive symptomatology (yes or no), diabetes prevalence (yes or no), hypertension prevalence (yes or no), hypercholesterolemia prevalence (yes or no), and cumulative average adherence to energy-restricted Mediterranean diet (low, median, or high). β-coefficients [95% CI] were estimated using linear regression models with robust standard errors to account for intracluster correlations. Significant values (*p*<0.05) were highlighted in bold type. Results were further adjusted for multiple comparisons using the Benjamini-Hochberg (BH) method.

**
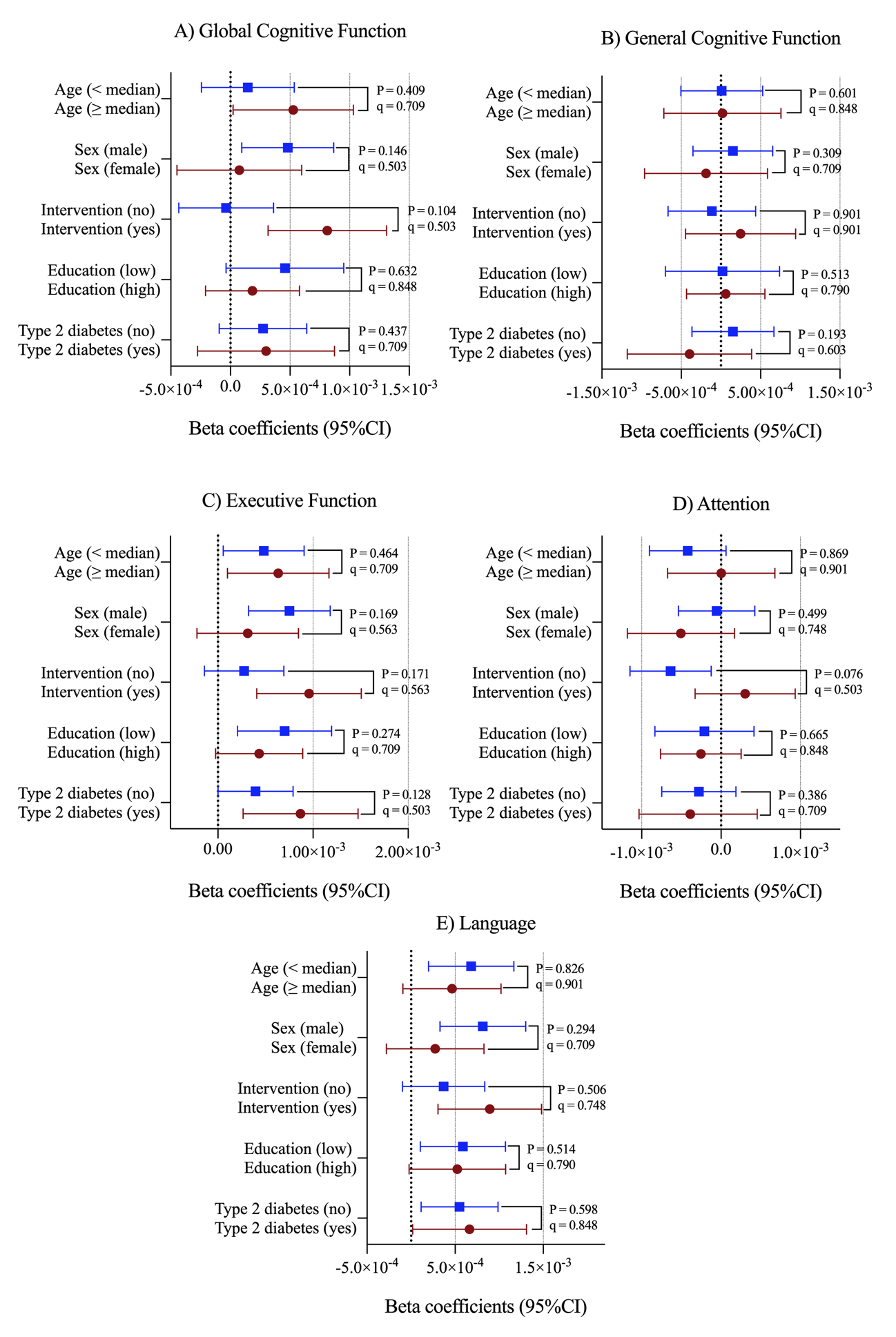
Figure S2.- Interaction between energy-adjusted cumulative average dietary betaine intake and different baseline variables of the study potentially related to cognitive function.** Abbreviations: BMI, body mass index; CI, confidence interval. Multivariable-adjusted models were adjusted for respective cognitive test score at baseline, age (years), sex, intervention group (control or intervention), recruiting center size (<200, 200-300, 300-450 or >450), education level (primary, secondary, or college), marital status (single, divorced or separated, married, or widower), body mass index (kg/m^2^), physical activity (metabolic equivalents in minutes per day), smoking status (current, former, or never), cumulative average of alcohol consumption in grams per day (and adding the quadratic term), depressive symptomatology (yes or no), diabetes prevalence (yes or no), hypertension prevalence (yes or no), hypercholesterolemia prevalence (yes or no), and cumulative average adherence to energy-restricted Mediterranean diet (low, median, or high). β-coefficients [95% CI] were estimated using linear regression models with robust standard errors to account for intracluster correlations. Significant values (*p*<0.05) were highlighted in bold type. Results were further adjusted for multiple comparisons using the Benjamini-Hochberg (BH) method.
