# Supplementary material for: Dietary Choline and Betaine Intake and 2-year Changes in Cognitive Function in Older Adults With Overweight or Obesity and Metabolic Syndrome: A Prospective Cohort Analysis
Source: Am J Clin Nutr. 2026 Mar 11;123(5):101265. doi: 10.1016/j.ajcnut.2026.101265 (PMC13197923; doi:10.1016/j.ajcnut.2026.101265)
Supplement: multimedia component 2 [file mmc2.docx]

**Supplementary methods**

A Spanish validated version of the Mini-Mental State Examination (MMSE) is a 30-point cognitive screening questionnaire with a value of rest-retest reliability of 0.87 (95%CI: 0.79-0.93) and convergent validity of -0.92 (correlation coefficient with the Alzheimer’s Disease Assessment Scale), which is divided into two sections (1,2). The first section requires vocal responses only examining different cognitive functions like orientation, memory, and attention. The second section tests the ability to name, follow verbal and written commands, write a sentence spontaneously and copy a complex polygon. A higher MMSE score indicates better cognitive performance.

The Clock Drawing Test (CDT) is another cognitive screening instrument mainly used to evaluate visuospatial and visuo-constructive capacities, as well as verbal and numerical knowledge, symbolic and conceptual representation, hemi-attention, memory, and executive function (including organization, planning and parallel processing) (3,4). The score ranges from 0 to 7 in the validated Spanish version used (5).

The Spanish Verbal Fluency Tests (VFTs) examine verbal ability and executive function, consisting of two parts: (i) the semantic verbal fluency task-animal category version (VFT-a), in which the participants were requested to name as many different animals as they can during 60 seconds; and (ii) in the phonemic verbal fluency task-letter “p” version (VFT-p), participants were asked to cite, in 60 seconds, as many words as possible that start with the letter P (avoiding names of people or places or repetitions of the same word with different suffixes). The total raw score for each of these tasks corresponds to the number of correct words produced (5–7).

The Digit Span Test (DST) of the WAIS-III Spanish version assesses attention and memory (5–8). The DST forward recall (DST-f), being representative of attention and short-term memory capacity, requires participants to repeat orally a series of random single digits in the same order as they heard, the sequence of digits varies in length from three to nine. The DST backward recall (DST-b), considered as a test of working memory capacity, requires participants to repeat a series of random single digits in reverse order, which the sequence varies from two to eight. The performance on the DST was reported by a direct score of the forward performance (ranging from 1 to 16) and the backward performance (ranging from 1 to 14).

The Trail Making Test (TMT), an instrument often used to assess executive function, consists of 25 circles spread out over two sheets of paper (parts A and B). In part A (TMT-A), which assesses attention and processing speed capacities, participants were requested to connect consecutive numbers (1–2–3–4-…) in the correct order by drawing a line. In part B (TMT-B), which further examines cognitive flexibility, participants were asked to connect consecutive numbers and letters in an alternating numeric and alphabetic sequence (1-A-2-B-3-C-…). Each part is scored according to the time taken to complete the task (a lower score represents better performance) (6,8–10).

**References:**

1. Folstein MF, Folstein SE, McHugh PR. “Mini-mental state”. A practical method for grading the cognitive state of patients for the clinician. J Psychiatr Res 1975;12:189–98.

2. Blesa R, Pujol M, Aguilar M, Santacruz P, Bertran-Serra I, Hernández G, Sol JM, Peña-Casanova J, NORMACODEM Group. NORMAlisation of Cognitive and Functional Instruments for DEMentia. Clinical validity of the “mini-mental state” for Spanish speaking communities. Neuropsychologia 2001;39:1150–7.

3. Shulman KI. Clock-drawing: is it the ideal cognitive screening test? Int J Geriatr Psychiatry 2000;15:548–61.

4. Aprahamian I, Martinelli JE, Neri AL, Yassuda MS. The Clock Drawing Test: A review of its accuracy in screening for dementia. Dement Neuropsychol 2009;3:74–81.

5. Rodríguez Laso A, García de Yébenes MJ, Frades Payo B, Bartolomé Martínez MP, Otero Puime Á, Sánchez Sánchez F, Ser Quijano T del. Evaluación cognitiva del anciano: Datos normativos de una muestra poblacional española de más de 70 años. Medicina clínica Doyma; 2004;122:727–40.

6. Peña-Casanova J, Quiñones-Ubeda S, Gramunt-Fombuena N, Quintana-Aparicio M, Aguilar M, Badenes D, Cerulla N, Molinuevo JL, Ruiz E, Robles A, et al. Spanish Multicenter Normative Studies (NEURONORMA Project): norms for verbal fluency tests. Arch Clin Neuropsychol 2009;24:395–411.

7. Patterson J. Multilingual Aphasia Examination. In: Kreutzer JS, DeLuca J, Caplan B, editors. Encyclopedia of Clinical Neuropsychology. [Internet] New York, NY: Springer; 2011 [cited 2024 May 8]. p. 1674–6. Available from: https://doi.org/10.1007/978-0-387-79948-3_900

8. de Jager CA, Dye L, de Bruin EA, Butler L, Fletcher J, Lamport DJ, Latulippe ME, Spencer JPE, Wesnes K. Criteria for validation and selection of cognitive tests for investigating the effects of foods and nutrients. Nutr Rev 2014;72:162–79.

9. Reitan RM. Trail Making Test: Manual for Administration and Scoring. Reitan Neuropsychology Laboratory; 1986. book p.

10. Llinàs-Reglà J, Vilalta-Franch J, López-Pousa S, Calvó-Perxas L, Torrents Rodas D, Garre-Olmo J. The Trail Making Test: Association With Other Neuropsychological Measures and Normative Values for Adults Aged 55 Years and Older From a Spanish-Speaking Population-Based Sample. Assessment SAGE Publications Inc; 2017;24:183–96.
